# Identification of clinically predictive metagenes that encode components of a network coupling cell shape to transcription by image-omics

**DOI:** 10.1101/gr.202028.115

**Published:** 2017-02

**Authors:** Heba Z. Sailem, Chris Bakal

**Affiliations:** 1 Institute of Cancer Research, Division of Cancer Biology, London SW3 6JB, United Kingdom

## Abstract

The associations between clinical phenotypes (tumor grade, survival) and cell phenotypes, such as shape, signaling activity, and gene expression, are the basis for cancer pathology, but the mechanisms explaining these relationships are not always clear. The generation of large data sets containing information regarding cell phenotypes and clinical data provides an opportunity to describe these mechanisms. Here, we develop an image-omics approach to integrate quantitative cell imaging data, gene expression, and protein–protein interaction data to systematically describe a “shape-gene network” that couples specific aspects of breast cancer cell shape to signaling and transcriptional events. The actions of this network converge on NF-κB, and support the idea that NF-κB is responsive to mechanical stimuli. By integrating RNAi screening data, we identify components of the shape-gene network that regulate NF-κB in response to cell shape changes. This network was also used to generate metagene models that predict NF-κB activity and aspects of morphology such as cell area, elongation, and protrusiveness. Critically, these metagenes also have predictive value regarding tumor grade and patient outcomes. Taken together, these data strongly suggest that changes in cell shape, driven by gene expression and/or mechanical forces, can promote breast cancer progression by modulating NF-κB activation. Our findings highlight the importance of integrating phenotypic data at the molecular level (signaling and gene expression) with those at the cellular and tissue levels to better understand breast cancer oncogenesis.

A tenet of genetics is that visually observable phenotypes can be used to infer the levels of unobservable biological properties, such as mRNA expression, protein levels/localization, and enzymatic activity. In the case of cells, quantifiable phenotypes such as cell shape can be used to infer the activation state of different signaling networks that regulate aspects of cell physiology, such as proliferation, survival, migration, and differentiation, even if the signaling activity of all these networks cannot be directly measured (Bakal et al. 2007; Sailem et al. 2014). Thus, visual phenotypes such as shape can be used to infer signaling states (Fig. 1A).

**Figure 1.**
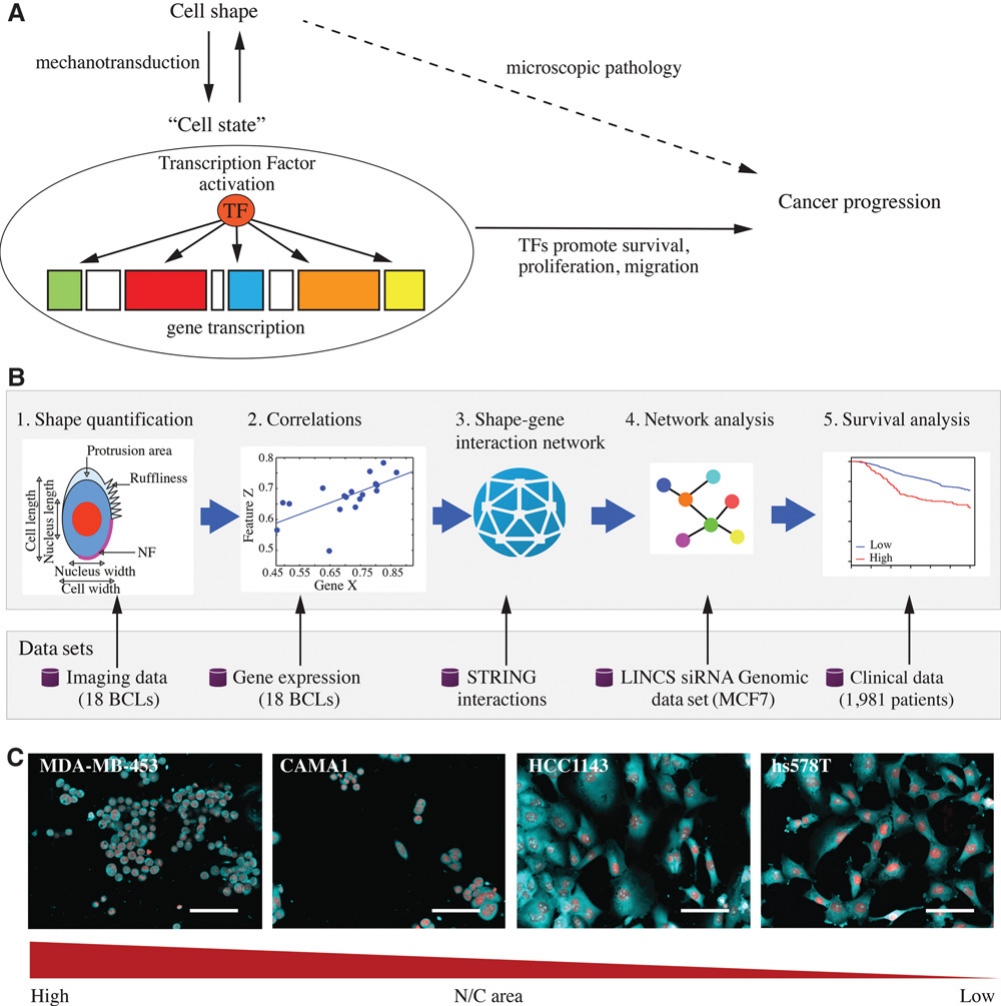
Integrating imaging and expression data. (*A*) The three-way relationship between cell shape, signaling states, and cancer progression. (*B*) Workflow for linking cell shape to transcription and patient outcome. (*C*) Representative images of different breast cancer lines (BCLs) to illustrate the variation in nucleus/cell area ratio (N/C area) MDA-MB-453, CAMA1, hs578T, and HCC1143 cells, where cell lines to the *left* have the highest N/C area and cell lines to the *right* have the lowest N/C area. Red: DAPI, cyan: DHE. Scale bar = 30 µm.

The relationship between cell shape and signaling states plays a role in the diagnosis and treatment of cancer. Even before the definition of a gene existed, observations were made that cancer cells have a different shape from normal cells and that disruptions in tissue architecture were symptomatic of cancer—findings which still remain a foundation of cancer pathology (Fig. 1A; Faguet 2015). It is now clear that the expression of oncogenes, or loss of tumor suppressors, affects numerous transcriptional, epigenetic, and post-translational processes, and thus signaling states, to promote cancer cell survival, proliferation, and invasion, and ultimately changes in cell shape (Fig. 1A; Simons et al. 1967). This three-way relationship between cell shape, signaling state, and clinical outcomes allows clinicians to make key decisions regarding patient treatment based partially on visual inspection of tumor tissue. While the relationship between signaling states and clinical outcomes is well understood—i.e., that oncogenic signaling can drive cancer—how cell shape is related to signaling is less clear. Most known relationships between the two properties are largely descriptive and qualitative in nature. Establishing quantitative and predictive relationships between cell shape and signaling states could increase the accuracy of patient diagnosis based on visually observable properties of tumor tissue.

A complicating factor in understanding the relationship between cell shape and signaling states is the bidirectional nature of this relationship (Fig. 1A). It is often assumed that the up-regulation of a gene and/or the activation of a protein results in cell shape changes—i.e., by altering cytoskeletal dynamics. However, in both stem and differentiated cells, cell shape regulates signaling and transcriptional activities (Orsulic et al. 1999; Miralles et al. 2003; Zheng et al. 2009; Olson and Nordheim 2010). In particular, shape regulates signaling via the actions of mechanosensitive components, including cell–cell adhesions, cell–matrix adhesions, and the cytoskeleton which can sense extracellular forces from the extracellular matrix (ECM), neighboring cells, and biological fluids (Mammoto et al. 2012). These mechanosensitive components, in turn, regulate cell shape and stiffness, as well as the signaling and transcriptional activities, in a process termed mechanotransduction. For example, changes in actin organization can affect the localization and activation of the YAP and TAZ transcription factors (TFs) (Yu and Guan 2013), and changes in the nuclear membrane structure can affect transcription via the action of lamins, which are both nuclear membrane scaffolds and TFs (Dahl et al. 2008). Therefore, to fully understand cancer initiation and progression, we need to develop methods for integrating information from cell shape as well as signaling states and transcriptional activities to study how these factors impact each other.

Previously, we have demonstrated that cell shape is a major regulator of the NF-κB signaling pathway in breast cancer cells using Bayesian learning-based methods (Sero et al. 2015). In normal cells, NF-κB regulates gene transcription in response to stress stimuli as a means to modulate the immune response, survival, proliferation, and tissue repair. NF-κB activity also plays a critical role in cancer progression, either by activating target genes in cancer cells and/or in infiltrating immune cells (Greten et al. 2004). Constitutive activation of NF-κB is characteristic of many cancers and as such there are intensive efforts to develop inhibitors of NF-κB signaling (Park and Hong 2016). Although activating mutations in the NF-κB signaling pathway are common in many lymphoid malignancies, they are rare in carcinomas (DiDonato et al. 2012); suggesting that NF-κB activation in solid tumors is driven in large part by extrinsic factors (Mantovani et al. 2008). While inflammation is clearly the primary activator of NF-κB in cancer cells, our work demonstrates that mechanical cues, such as those coming from the loss of cell–cell adhesions, or increases in cell autonomous contractility, can also up-regulate NF-κB activity via changes in cell shape (Sero et al. 2015). Because tumor cells often lose cell–cell adhesion, and remodel their microenvironment in a way that results in both increased stiffness and deposition of ECM which increases cellular contractility (Butcher et al. 2009), mechanical activation of NF-κB could represent an important means by which NF-κB is extrinsically activated in cancer cells.

## Results

### Identifying genes that correlate with cell shape features in breast cancer cells

To describe the signaling networks that couple cell shape to transcriptional regulation, we identified genes whose expression correlates with differences in cell shape across different breast cancer lines (BCLs) (Fig. 1B). We speculated that these genes should either regulate cell shape and/or act as part of mechanotransduction pathways that alter gene expression in response to changes in cell shape. To generate these networks, we made use of (1) a data set where we measured shape features in 307,643 cells across 18 BCLs (Supplemental Table S1; Sailem et al. 2015; Sero et al. 2015), and (2) a data set describing the expression of 28,376 genes across these same 18 BCLs (Grigoriadis et al. 2012). Ten morphological features that we have previously shown to be predictive of TF activation were used in our analysis (Sero et al. 2015). These include: the dimensions and area of the cell and the nucleus; nucleus/cell area ratio (N/C area); centers distance (the distance between the cell center and the nucleus center); neighbor fraction (NF—the fraction of cell border in contact with other cells); cellular protrusions (areas at the periphery of the cell with lower intensity than the rest of the cell, reflecting thinner cellular regions); and cell ruffliness (variation in membrane region intensity) (Fig. 1B,C).

Cell shape features were integrated with gene expression data by measuring the correlation between the average and the standard deviation (SD) of the morphological features and the expression of each gene (Fig. 1B). Genes with low variability across BCLs were filtered out, resulting in 11,314 genes that were used in the analysis (Methods). We found 504 genes (termed shape-correlated genes hereafter) to be significantly correlated with the morphological features (absolute Spearman correlation > 0.7, *P*-value < 0.0012, False Discovery Rate [FDR] < 23%) (Supplemental Figs. S1, S2; Supplemental Table S2).

### Morphological features can be linked to genes that regulate cell shape at the molecular level

To investigate whether different shape-correlated genes encode components which regulate specific cellular processes, we performed enrichment analysis of the 504 genes using Gene Set Enrichment Analysis (GSEA) (Subramanian et al. 2005) and DAVID (Huang et al. 2009). We only considered terms enriched at an FDR *P*-value < 0.05. Shape-correlated genes are enriched for cellular processes associated with cell morphogenesis, especially those involved in differentiation and cell migration (Supplemental Table S3). Additionally, shape-correlated genes are enriched for adhesion-related processes such as cell adhesion, ECM organization, ECM-receptor interaction, focal adhesion, and cell projection morphogenesis (Supplemental Table S3). The enrichment of these categories strongly supports the idea that these genes are either regulated by or regulate changes in cell shape.

We also performed enrichment analysis for genes correlated with each shape feature individually (versus enrichment analysis of all 504 genes simultaneously). For example, cell elongation, as measured by cell-width/cell-length ratio (cell W/L), correlates with 135 genes that are involved in processes that ultimately affect cell elongation, such as cell cytoskeleton (six genes), regulation of cell proliferation (eight genes), and cell adhesion (nine genes) (Supplemental Table S4). Seven of the 86 genes that correlate with N/C area are in the KEGG “focal adhesions pathway,” and eight genes are categorized by gene ontology as ECM components (Supplemental Table S4). This analysis also resulted in unexpected links between genes and some phenotypic features. For example, 10 of the 43 genes that correlate with nuclear roundness encode mitochondrial components (Supplemental Table S4). Using GSEA analysis, we also found that 20 of N/C area-correlated genes are shown to be down-regulated in luminal-like cell lines, such as CAMA1 and MDA-MB-453, versus mesenchymal-like cell lines, such as HCC1143 and hs578T (Fig. 1C), including the genes *HMGA2*, *PIK3CD* and *VCL*. By integrating gene expression data with phenotypic data of cell lines from the same tissue, we are able to link morphological features to the expression of specific genes.

### Building a shape-gene interaction network

The biggest intra- and inter-cell line shape differences in this data set can be linked to epithelial-like or mesenchymal-like morphologies. Thus, we were particularly interested in understanding how phenotypic features and shape-correlated genes interact with a set of TFs that are involved in epithelial-mesenchymal transition (EMT), such as RELA (the p65 subunit of NF-κB), SMAD2, SMAD3, SNAI1, SNAI2, TWIST1, ZEB1, and ZEB2 (Garg 2013; Bogachek et al. 2014; Puisieux et al. 2014). As mesenchymal cells have been described to have stem-like properties (Samavarchi-Tehrani et al. 2010), we also investigated interactions between genes in these data and TFs that regulate stemness and differentiation, such as KLF4, MYC, and SOX2 (Li et al. 2010; Samavarchi-Tehrani et al. 2010). Finally, we also determined the relationship between shape-correlated genes and the mechanosensitive TF YAP1, which has roles in cell proliferation and EMT (Yu and Guan 2013). Of these preselected genes, only *SMAD3* expression correlates with breast cancer cell shape; specifically, *SMAD3* expression correlates with cell W/L (Supplemental Fig. S1; Supplemental Table S2). That the expression of the other selected TFs does not correlate with cell morphology is not unexpected because TF activity is often regulated by post-translational mechanisms, such as subcellular localization and/or phosphorylation.

**Figure 2.**
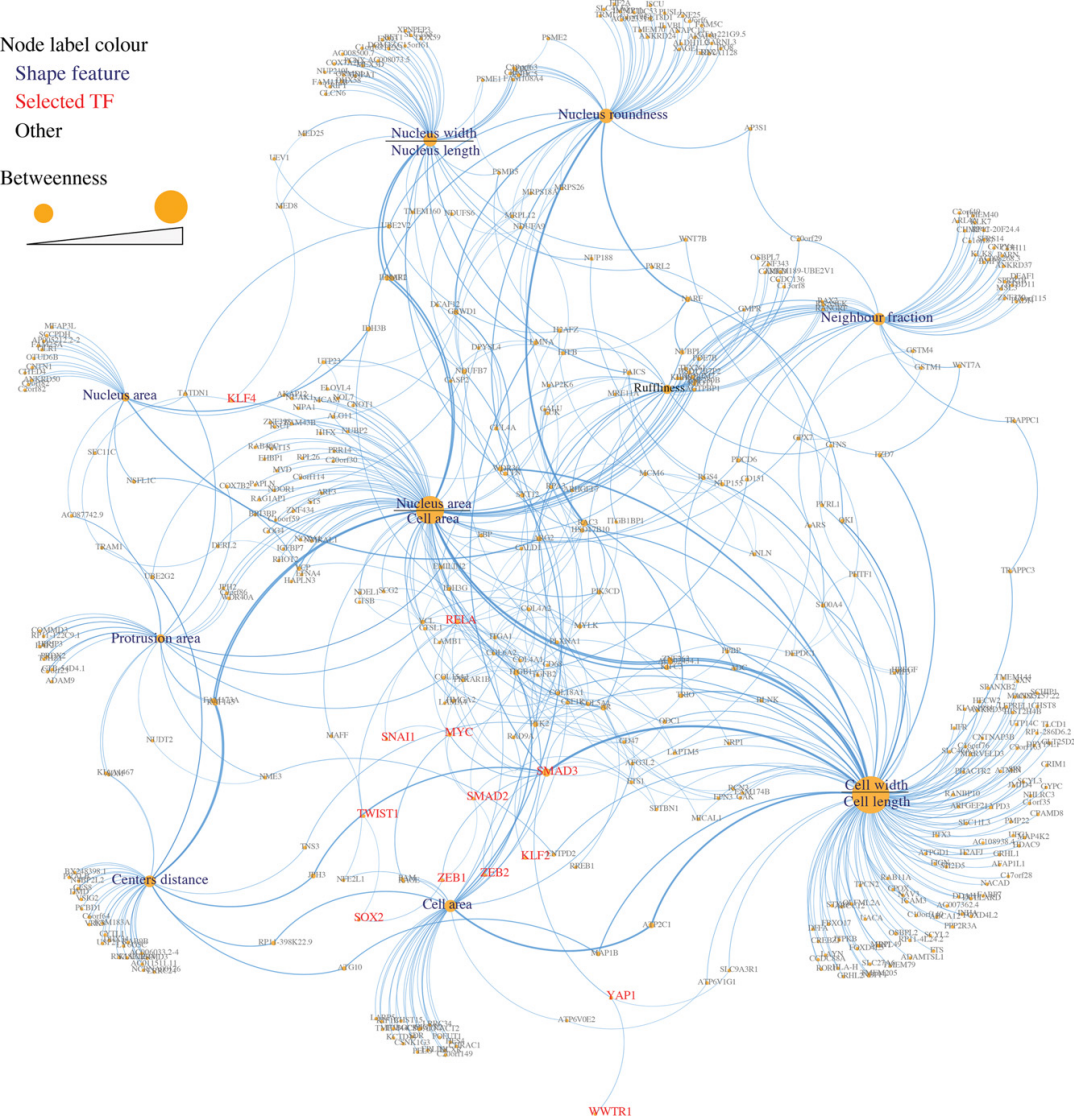
Shape-gene interaction network. A network of the interactions between the proteins encoded by shape-correlated genes, selected TFs, and shape features. Node size and font size represent the betweenness of a node, which reflects the centrality of the node.

We used the STRING database (Jensen et al. 2009) to retrieve interactions between proteins encoded by shape-correlated genes and between proteins encoded by shape-correlated genes and the selected TFs. We considered STRING interactions that were of medium confidence (combined STRING score ≥ 0.4), where the combined score is calculated based on known experimentally derived and curated interactions, as well as predicted interactions based on neighborhood, gene fusions, co-occurrence, and co-expression (Methods). This resulted in 210 interactions, 22 of which are between shape-correlated proteins and our selected TFs as follows: SMAD3 (eight interactions), RELA (six), MYC (four), KLF4 (two), SMAD2 (one), and YAP1 (one) (Supplemental Table S5). In addition to interactions between proteins encoded by shape-correlated genes, we added shape feature-gene correlations as interactions (514 edges). The resulting list of interactions was used to build a shape-gene interaction network that shows how morphological features are linked to different genes and selected TFs (Fig. 2).

### Network analysis

**Figure 3.**
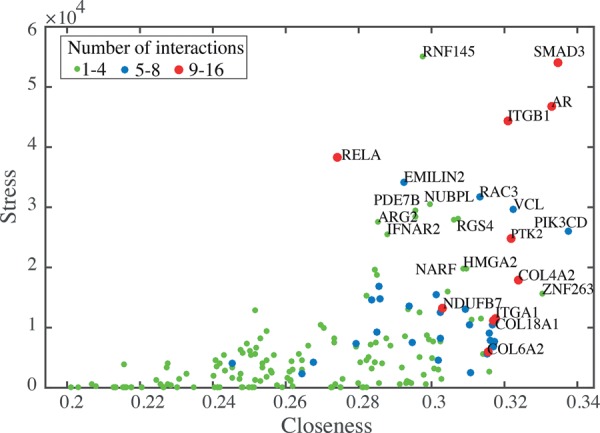
Analysis of the shape-gene network. A plot of the properties of gene nodes summarizing degree, closeness, and stress. Protein names for nodes that have high values for any of these features are shown.

We analyzed the main attributes of nodes in the shape-gene network including node degree, stress, and closeness. Node degree is the number of node interactions with other nodes (between one and 16 in our network) (Fig. 3). The stress of a node is determined by calculating the number of shortest paths that span a node and reflect the activity of the node (Shimbel 1953). The closeness of a node represents the reciprocal of the average length of shortest paths that span across the node and indicates how fast information can spread from that node through the network (Newman 2005). Interestingly, we found that many cell-ECM adhesion nodes, including ITGB1, ITGA1, COL6A2, COL18A1, PTK2, and VCL, have high degree and closeness values (Fig. 3). This suggests that mechanical signals, such as changes in adhesion/shape, received by these nodes are rapidly propagated throughout the network. We also observed that the TFs SMAD3, Androgen Receptor (AR), and RELA have high node degree and stress values compared to all other nodes in the network (and not just other TFs), suggesting that these TFs are highly active in coordinating the actions of multiple network components. That we identified a central role for RELA/NF-κB in this network in a largely unsupervised manner is consistent with our previous observation that NF-κB activity is regulated extensively by cell shape (Sero et al. 2015).

As RELA and SMAD3 nodes have particularly high degree and stress scores, we sought to determine the shortest paths that exist between each of the shape features and these TFs (Methods; Supplemental Fig. S3). These paths represent potential interactions linking cell shape to transcription. YAP1 was also included in the analysis for comparison, because it is a well-known mechanoresponsive protein. We only considered direct paths that do not involve other phenotypic features or other preselected TFs (Supplemental Table S6; Supplemental Fig. S3A,B) and considered both optimal, as well as suboptimal, paths (Methods). Nodes in these paths are either mechanosensitive genes that possibly regulate SMAD3 and RELA directly or indirectly or are regulated by SMAD3 and RELA and are thus mechanoeffector genes.

**Figure 4.**
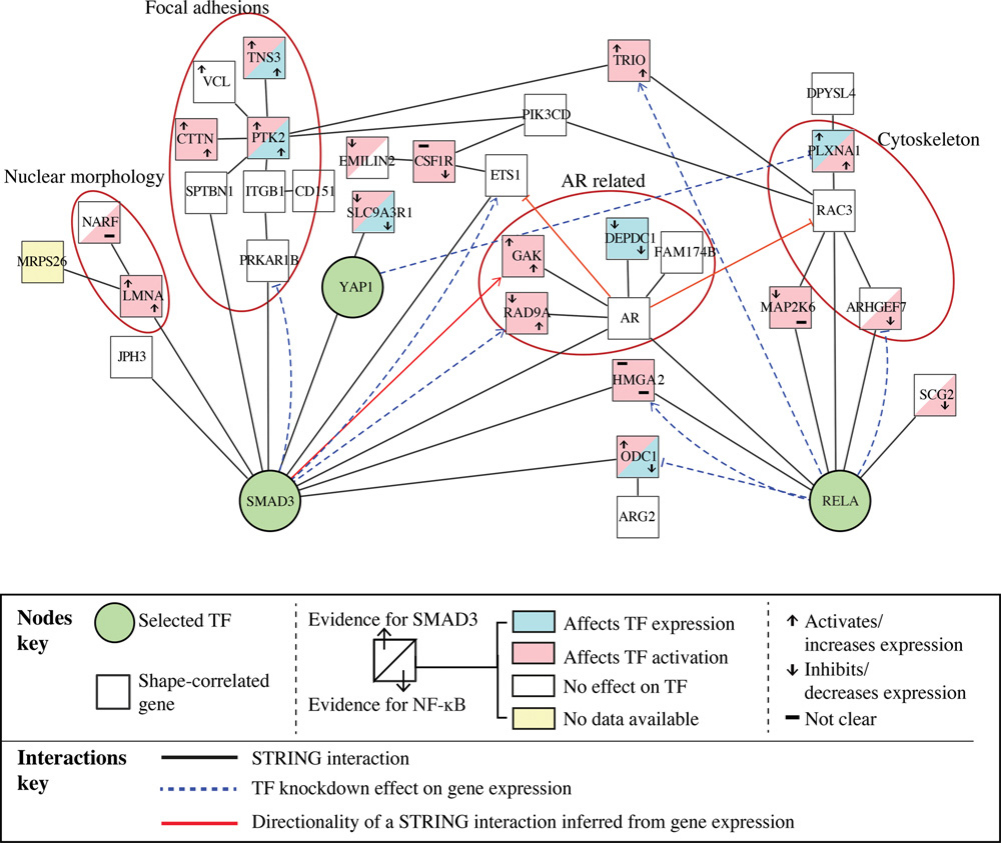
SMAD3-NF-κB subnetwork. Proteins that are in a direct path from a phenotypic feature to RELA, SMAD3, or YAP1 (Supplemental Table S6) and their interactions based on STRING. Edges in dashed lines are based on gene expression and indicate feedback from a TF to proteins encoded by shape-correlated genes.

From the interactions in the path analysis, we built a SMAD3-NF-κB subnetwork (Fig. 4; Supplemental Table S6). In this network, SMAD3 activation is linked to both a “nuclear morphology” module (containing LMNA) and a “focal adhesion” module (containing PTK2 and TNS3). An AR module (containing AR, GAK, RAD9A, DEPDC1, and FAM174B), as well as TRIO, HMGA2, and ODC1 gene nodes, are linked to both SMAD3 and RELA. Thus, the architecture of this network depicts how nuclear shape, adhesion, and cytoskeleton can be linked to SMAD3 and NF-κB signaling.

### Predicted regulators of SMAD3 and RELA activation

We sought to identify mechanosensitive nodes in the SMAD3***-***NF-κB subnetwork that regulate the activation and transcriptional activity of SMAD3 and RELA versus those that might regulate the mRNA levels of each TF (Supplemental Fig. S3C). To perform the analysis, we used the Broad Institute's Library of Integrated Network-based Cellular Signatures (LINCS) RNAi data set that describes the expression of 3287 essential genes in MCF7 breast cancer cells after 22,268 gene knockdowns (Duan et al. 2014). To predict whether a shape-correlated gene node in the SMAD3-NF-κB subnetwork is a regulator of SMAD3 or RELA, we identified gene nodes in the network whose depletion affects the expression of downstream targets of SMAD3 or RELA (absolute *z*-score = 1.5) without changing the expression of the TF itself (Methods). We also identified gene nodes whose depletion affects TF mRNA expression (i.e., expression of *RELA* and *SMAD3* mRNA). A protein was considered to affect the TF activation significantly if its depletion affects the expression of at least 10% of the TF targets and the overlap between the protein's putative target genes and the TF's target genes is statistically significant using hypergeometric probability (*P*-value < 0.05) (Supplemental Table S7). We further estimated whether proteins encoded by shape-correlated genes promote or suppress TF activity by determining whether the depletion of the protein affects the expression of TF targets in a similar way as TF knockdown (activators) or has the opposite effect (suppressors) (Methods). For example, knockdown of *TRIO* significantly changes the expression of 518 genes of which 134 genes were identified as SMAD3 targets and 112 were identified as RELA targets (*P*-value ≤ 0.01). Since *TRIO* knockdown affects RELA and SMAD3 targets in similar ways as the TF knockdowns, we predict that TRIO activates SMAD3 and RELA (Fig. 4). Using this method, we found that many of the nodes in Figure 4 are RELA and SMAD3 activators/inhibitors (i.e., through post-translational mechanisms) and identified nodes whose activity regulates *RELA* and *SMAD3* expression. Because the expression of these genes correlates with cell shape, these are likely mechanosensitive genes that regulate SMAD3 and/or RELA activation in response to mechanical cues.

### SMAD3 and RELA targets that correlate with cell shape

We determined whether any of the shape-correlated genes in the SMAD3-NF-κB subnetwork were changed after *SMAD3*, *RELA*, or *YAP1* knockdown and thus could be considered mechanoeffector targets of these TFs (Methods). We found that *SMAD3* knockdown significantly increases expression of the *RAD9A* and *ETS1* TFs, while it significantly decreases *PRKAR1B* expression (absolute *z*-score > 1.5). *RELA* knockdown significantly increases *TRIO* and *HMGA2* expression and decreases *ODC1* and *ARHGEF7* expression (Fig. 4). *YAP1* knockdown significantly decreases *PLXNA1* expression (Fig. 4). These results suggest that components of the network such as TRIO and RAD9A regulate SMAD3 and/or RELA activity, which in turn regulate ARHGEF7, ETS1, ODC1, HMGA2, PRKAR1B, RAD9A, and TRIO via feedback loops. The expression of these target genes, especially *ARHGEF7* and *TRIO*, is likely to change cell shape (Moshfegh et al. 2014).

### Genes regulating SMAD3-NF-κB subnetwork are differentially expressed in different BCL molecular subtypes

**Figure 5.**
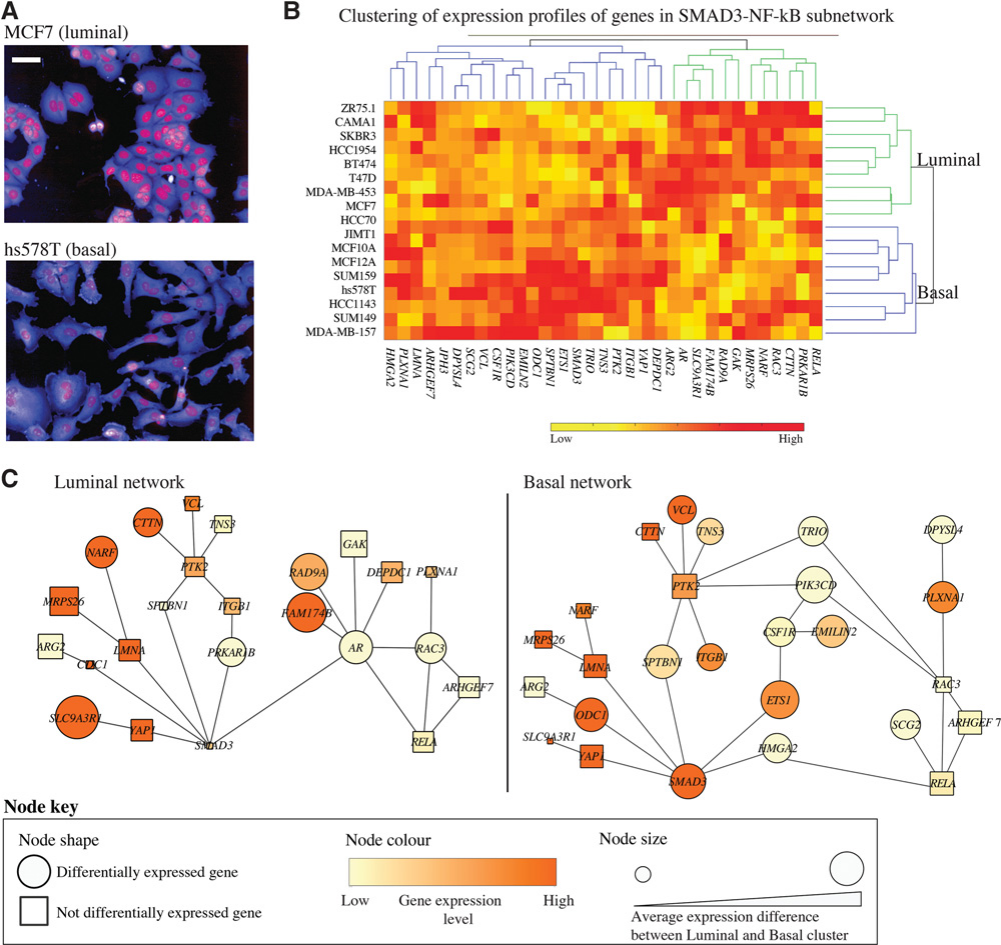
The expression profiles of shape-correlated genes that drive transcriptional activities of SMAD3 and RELA in luminal versus basal breast cell types. (*A*) Representative examples of luminal vs. basal shapes. Red: DAPI, blue: DHE. Scale bar = 50 µm. (*B*) Clustering of 18 BCLs based on the expression of shape-correlated genes in Figure 4 separates cell lines into luminal (green) and basal (blue) subtypes. The basal cluster includes only basal cell lines. The luminal cluster includes mostly luminal cell lines and the basal A cell lines HCC70 and HCC1954. (*C*) Networks of the expressed genes in the luminal/basal clusters in *B*, where differentially expressed genes between luminal and basal clusters are represented as circles. Genes that have a higher average expression difference between luminal and basal cluster have a larger node size. Node color indicates the average expression values in each cluster.

We and others have shown that different BCL molecular subtypes have distinct cell morphologies, where luminal cell lines adopt primarily epithelial shapes, while basal cell lines adopt mesenchymal shapes (Fig. 5A; Neve et al. 2006; Sero et al. 2015). Clustering of expression profiles of the genes in the SMAD3-NF-κB mechanosensitive subnetwork reveals two main clusters of BCLs that also correlate with BCL luminal and basal subtypes (Fig. 5B). We rederived SMAD3-NF-kB subnetworks to highlight the difference in transcriptional activities between luminal and basal cells (Fig. 5B,C; Methods). These networks reveal that SMAD3 activity is likely to be minimal in luminal BCLs, while the activity of the AR module is very high. Interestingly, the expression of *SLC9A3R1,* encoding a protein that sequesters YAP1 in the cell membrane (Mohler et al. 2015), is highly expressed in the luminal network (Fig. 5C). On the other hand, *SMAD3*, *ETS1*, and *HMGA2* expression is high in basal cells, which is consistent with their role in EMT. *RELA* is not differentially expressed in basal versus luminal BCLs, although RELA localization has been shown to correlate with BCL molecular subtypes (Sero et al. 2015). Taken together, we propose that the activity of the SMAD3-NF-κB subnetwork, which acts in response to, and drives, cell shape changes, may be responsible for a number of phenotypic differences between these cell types.

### Derivation of morphological metagenes

We next sought to determine if the signaling state of the BCL shape-gene network in Figure 2 contributes to the progression of breast cancer in patients. Therefore, we investigated if the expression of genes encoding components of this network that correlate with a specific shape feature also correlate with diagnostic and/or clinical outcome. This approach allows us to leverage the “three-way” relationship between shape, signaling state (as determined by gene expression), and disease progression and to overcome the issue that, while many generated patient data sets contain information about tumor grade, patient outcomes, and gene expression, they do not contain information regarding single-cell shape.

We derived multilinear regression models that estimate the value of a morphological feature or the SD of that feature based on the expression of a selected subset of genes that correlate with that feature (Supplemental Tables S8,S9; Methods). We termed the model predictions of morphological features based on gene expression “morphological metagenes,” which can be considered as a weighted sum of the expression of genes that correlate with that morphological feature. For example, the cell area SD metagene is described by the model

Cell area SD=0.23×CHRAC1+0.25×LARP4B−0.15×CHST15+1.21.

**Figure 6.**
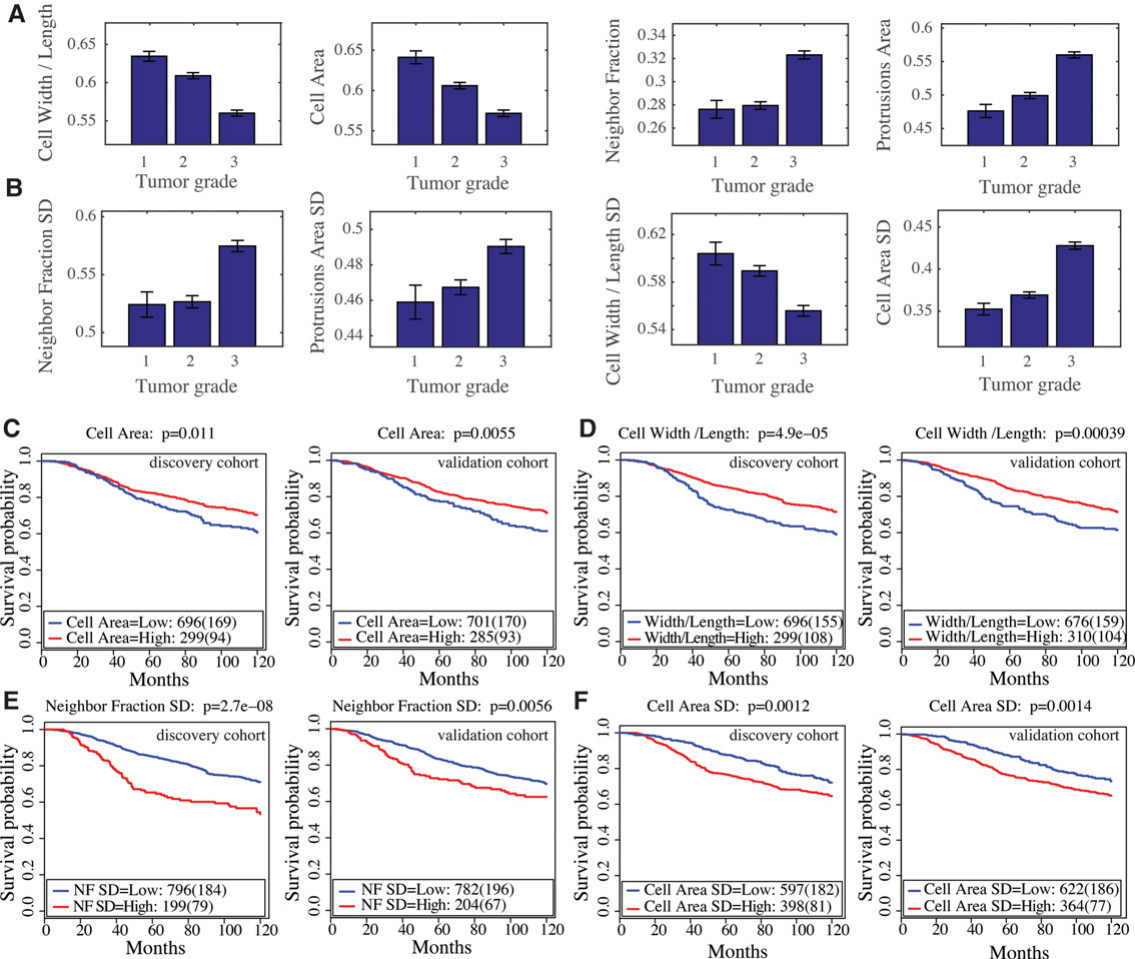
The prognostic value of the morphological metagenes and their associations with the clinical parameters in the METABRIC data set. (*A*) Association between tumor grade and cell W/L, cell area, NF, and protrusion area metagenes. All these associations are significant, with *P*-value < 0.0005 using the Jonckheere–Terpstra test. Error bars indicate the standard error of the mean (SEM). (*B*) Association between tumor grade and NF SD, protrusion area SD, cell W/L SD, and cell area SD metagenes. These associations are significant, with *P*-value < 0.0005 using the Jonckheere–Terpstra test. Error bars indicate the SEM. (*C–F*) Kaplan–Meier curves to illustrate the disease-specific survival probabilities of patient groups in discovery and validation cohorts in the METABRIC data set stratified by (*C*) cell area, (*D*) cell W/L, (*E*) NF SD, and (*F*) cell area SD metagenes.

To investigate the in vivo relevance of our metagenes, we used breast cancer patient data from the Molecular Taxonomy of Breast Cancer International Consortium (METABRIC). This data set includes expression profiles, clinical features, and disease-specific survival for 1981 breast cancer patients; 995 patients in the discovery cohort and 986 patients in the validation cohort (Curtis et al. 2012). We found that the expression of different metagenes correlates with tumor grade that is based on the extent of cell differentiation and invasion (Fig. 6A,B; Elston and Ellis 1991). For example, we found that cell W/L and cell area metagenes are negatively correlated with tumor grade (Jonckheere–Terpstra test *P*-value < 0.0005) (Fig. 6A). Because high values of cell W/L metagene are indicative of epithelial shape, while lower values are indicative of a more mesenchymal shape (Zhao et al. 2016), this suggests that genes associated with epithelial shapes are down-regulated in highly aggressive breast cancers. Moreover, this supports the idea that the activity of the shape-gene network contributes to disease progression.

Additionally, the expression of the NF metagene, which predicts local cell density, and hence, proliferation rate (Snijder et al. 2009), and the protrusion metagene, are significantly higher in grade 3 tumors (*P*-value < 0.0001) (Fig. 6A). That genes associated with high NF and protrusions have high expression in more aggressive tumors is in line with the idea that aggressive tumors have regions of high cell density driven by high rates of proliferation and have protrusive invasive fronts (Jögi et al. 2012; Zhao et al. 2016).

Metagenes that predict variability in cellular morphology in BCLs also correlate with tumor grade. The NF SD, protrusion area SD, and cell area SD metagenes correlate positively with tumor grade (*P*-value < 0.0005) (Fig. 6B). On the other hand, the cell W/L SD metagene correlates negatively with tumor grade (Fig. 6B).

To validate the significance of the correlation between the expression of morphological metagenes and clinical data, we derived a random variable and built a regression model to predict this variable from 20 randomly drawn genes, as we have done for the other metagenes (Methods). As expected, the random metagene does not correlate with tumor grade (*P*-value = 0.2698).

### Prognostic value of morphological metagenes

We also determined whether morphological metagenes could stratify patients based on 10-yr patient-specific survival. To dichotomize our metagenes, we selected the cut-offs that produce the best prognostic predictions based on the discovery cohort (Methods; Nawaz et al. 2015). Patients with high values of the cell area and cell W/L metagenes have significantly better survival in both the discovery and validation cohorts (log-rank test *P* < 0.01) (Fig. 6C,D). Patients with high values of NF SD metagenes, which indicates high variation in cell density, have significantly worse prognosis (log-rank test *P* < 0.0056) (Fig. 6E). Furthermore, tumors with high expression of the cell area SD metagene also have a worse prognosis (log-rank test *P* < 0.0014) (Fig. 6F).

We performed a multivariate Cox proportional hazards model to identify whether these metagenes can provide independent prognostic factors from other clinical factors including tumor size, grade and lymph node status. We found that the cell W/L and NF SD metagenes significantly predict prognosis independent of tumor size and existence of lymph nodes (*P*-value < 0.05 in both discovery and validation cohorts) but not tumor grade (Supplemental Table S10). Interestingly, only the cell area SD metagene significantly predicts prognosis independently of tumor size, grade, and node status (*P*-value < 0.05 in both discovery and validation cohorts) (Supplemental Table S10). These results further illustrate that our morphological metagenes recapitulate the grading performed by pathologists and suggest a prognostic value of the cell area SD metagene. These data further support the idea that the activity of the shape-gene network contributes to breast cancer progression in patients.

### NF-κB activation metagene

**Figure 7.**
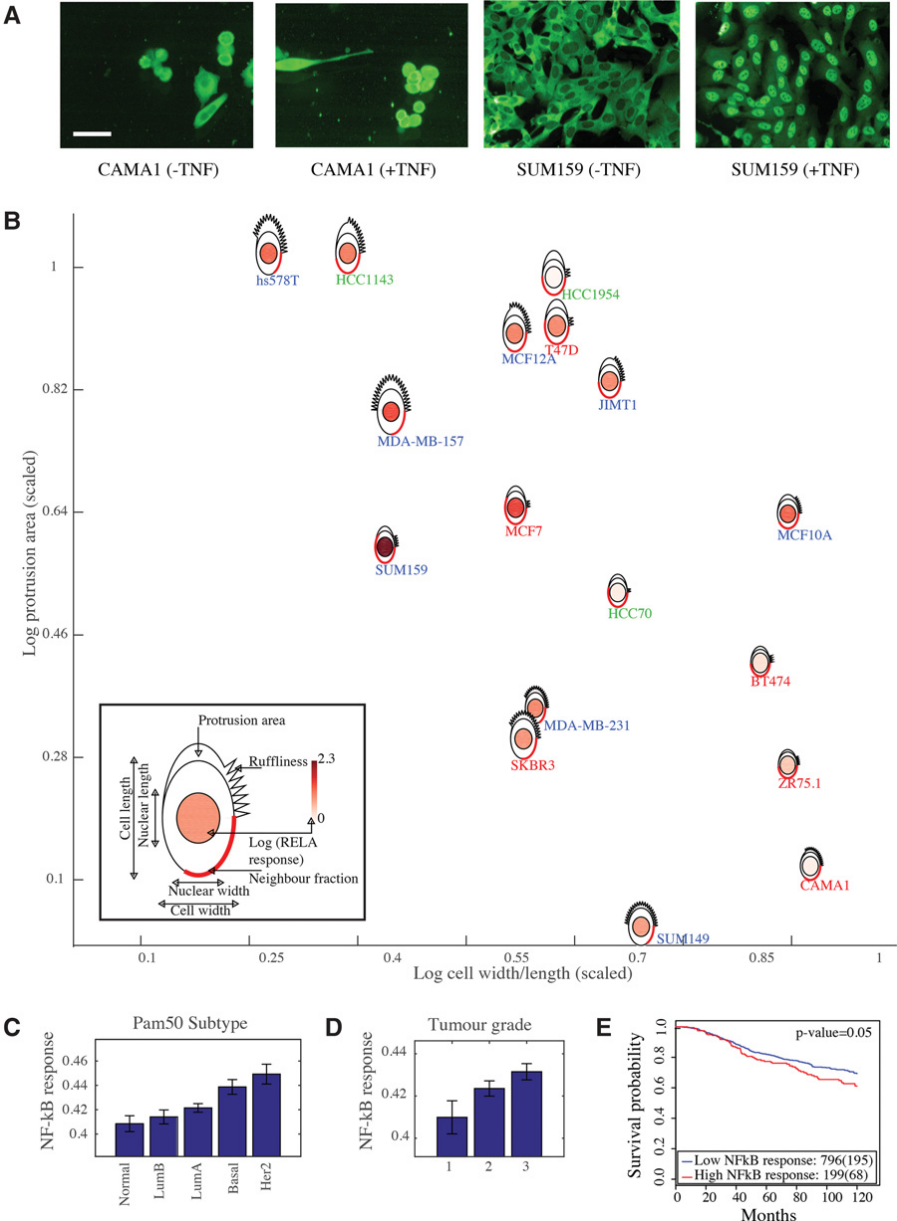
Derivation of NF-κB response metagene and its association with the clinical parameters in the METABRIC data set. (*A*) CAMA1 and SUM159 cells stained with anti-RELA/NF-κB antibody (−/+TNF). Scale bar = 50 µm. (*B*) Representation of seven morphological BCL features and RELA response (fold change +TNF/−TNF) using PhenoPlot (Sailem et al. 2015), where BCL glyphs are positioned based on the value of cell W/L (*x*-axis) and protrusion area (*y*-axis). Cell line label color indicates molecular subtype. Red: luminal, green: basal A, and blue: basal B. (*C*) Association between Pam50 subtype and NF-κB response metagene (Jonckheere–Terpstra test *P*-value < 0.0005). Error bars indicate the SEM. (*D*) Association between tumor grade and NF-κB response metagene (Jonckheere–Terpstra test *P*-value < 0.0005). Error bars indicate the SEM. (*E*) Kaplan–Meier curves to illustrate the disease-specific survival probabilities of patient groups in the discovery cohort in the METABRIC data set, stratified by NF-κB response metagene.

Because metagenes encoding components of the shape-gene network are predictive of clinical outcomes and of the central position of RELA/NF-κB within this network, we reasoned that NF-κB activity may play a role in cancer progression in response to the actions of this network. For example, changes in cell shape may affect the activity of the shape-gene network, and thus NF-κB activity, to promote tumor cell proliferation, survival, and invasion. To test this hypothesis, we defined a metagene that correlates with RELA activation in response to TNF across 18 BCLs which is measured as the ratio between nuclear RELA intensity and cytoplasmic RELA intensity (Methods; Fig. 7A; Sero et al. 2015). The response of RELA to TNF is defined as the log of the average RELA ratio (+TNF) divided by the average RELA ratio (−TNF). To build a regression model of the NF-κB response metagene, we used BCLs expression data of genes that affected either RELA activation or expression in Figure 4 (Supplemental Table S11; Methods). We found a significant association between the NF-κB response metagene and Pam50 subtype (*P*-value < 0.0005), which groups patients into luminal A, luminal B, HER2, basal, and normal subtypes (Sorlie et al. 2001). Interestingly, the expression of the NF-κB response metagene is higher in basal and HER2 tumors compared to luminal tumors, which is consistent with our 2-D data (Fig. 7B,C).

The NF-κB response metagene also correlates with tumor grade (*P*-value < 0.0005) (Fig. 7D). Furthermore, the NF-κB response metagene predicts patient survival, although this is only significant in the METABRIC discovery cohort (*P*-value < 0.05) (Fig. 7E). These results provide in vivo support for our previous finding that NF-κB signaling is modulated by cell shape (Sero et al. 2015), which may drive cancer cell survival, proliferation, and invasion (DiDonato et al. 2012). Furthermore, these results suggest that changes in the activity of the shape-gene network can impact NF-κB activation to drive disease.

## Discussion

The accumulation of large data sets describing cell shape, gene expression, and tumor phenotypes provides a starting point from which to better quantify the three-way relationship between cell shape, signaling states, and cancer prognosis. However, there are still several challenges in integrating these data sets. In particular, it remains technically challenging to collect such data sets on the same cells. Moreover, different omic data sets are collected at different levels. Imaging data sets might define cell shape using hundreds of features for millions of single cells; gene expression data sets typically contain the average gene expression for thousands of genes in a population; and clinical data sets might contain gene expression and outcome data for hundreds of patients but have very little data concerning cancer cell shape. Here, we developed a method that overcomes many of these challenges by leveraging different data sets of breast cancer cells that have been quantitatively imaged and expression-profiled in parallel to generate a shape-gene network. This network can be used not only to gain insights into the interaction between cell shape and transcription via the actions of different signaling pathways but can also be used to derive metagenes that have clinically predictive value.

For decades, pathologists have diagnosed tumors based on phenotypes, such as nuclear morphology and differentiation status of cells from hematoxylin- and eosin-stained tumor images, where tumors with a more normal epithelial organization are given lower grades, while tumors with a less epithelial organization are given higher grades. Indeed, our morphological metagene models suggest that cancer cells in higher tumor grades are more elongated and protrusive, which is consistent with the idea that cells in these tumors are more mesenchymal-like (Mani et al. 2008). We also found that cell area, NF, and protrusiveness SD metagenes correlate positively with tumor grade, while the cell W/L SD metagene correlates negatively with tumor grade. One implication of these findings is that phenotypic heterogeneity in terms of proliferation and protrusion correlates positively with poor prognoses, but heterogeneity in terms of cell shape (as measured by W/L) does not. We speculate that such variation in cell W/L may reflect the fact that less aggressive tumors still retain some aspect of normal mammary tissue architecture, such as the presence of both myoepithelial (more elongated) and luminal cells (less elongated). More studies are required to validate the extent to which these morphological metagenes recapitulate the morphology of in vivo tumor cells. Nonetheless, based on the consistency of our findings with the literature, we propose that the derivation of morphological metagenes allows us to infer tumor cell shape and phenotypes and to effectively bridge the gap between cell shape and patient outcomes that is presented by the lack of shape data in clinical data sets.

A limitation of our metagenes is that they are based on 2-D cell shape data that might not reflect the whole variation of cell shape in a 3-D tissue environment. However, we believe that imaging of cells in 2-D is particularly advantageous as it enables us to generate high-quality quantitative phenotypic signatures of relatively homogenous cancer cell populations, allowing us to make strong correlations between signaling states and specific cell shape features. Moreover, even as single-cell phenotyping technology continues to evolve (Lee et al. 2014), linking shape, signaling states, or gene expression to cancer progression by analyzing single cancer cells in tumors will still be challenging because of the complex 3-D architecture of tumors (Egeblad et al. 2010).

We have previously shown that NF-κB activity is regulated by breast cancer cell shape in 2-D cell culture conditions. In particular, cell protrusiveness, cell spreading, nuclear shape, and cell–cell contact can predict levels of RELA translocation (Sero et al. 2015). Moreover, we have demonstrated that cell-to-cell differences in shape can lead to sharp gradients of RELA activity in a tissue, such as between mesenchymal-like cells at the edge of a wound and epithelial cells completely surrounded by other cells (Sero et al. 2015). However, the mechanisms by which cell shape is linked to NF-κB signaling and whether shape-mediated regulation has a role to play in cancer remained unclear. By systematically analyzing the interactions between shape-correlated genes and key EMT TFs, we confirm that NF-κB, as well as SMAD3, play a major role in sensing shape information in BCLs. Through the analysis of transcriptional profiles following systematic knockdowns of the components of this network, we are able to classify particular components as regulators or effectors of NF-κB signaling and thus classify these genes as mechanosensitive or mechanoeffectors, respectively. Importantly, we show that the expression of metagenes explaining RELA translocation in tissue culture correlates with poor prognosis in vivo, which strongly suggests that the actions of this shape-gene network converge on NF-κB to drive tumorigenesis and potentially metastasis. We believe that this convergence largely explains why other shape metagenes (i.e., protrusion) also correlate with poor patient outcomes, although we cannot exclude the possibility that the expression of shape metagenes regulates the activity of other TFs (especially SMAD3) or has post-transcriptional effects on tumorigenesis. One implication of this work is that NF-κB activity can be driven by mechanical and geometric cues in breast tumor microenvironments even in the absence of activating mutations in genes encoding components of the NF-κB pathway. The role of mechanical and geometric cues in regulating NF-κB, in addition to the inflammatory nature of many tumors, may in part explain why mutations of this pathway are relatively infrequent in solid carcinomas, even though NF-κB has been shown to be a common driver of disease (DiDonato et al. 2012).

That cell shape can regulate signaling states has important implications for our understanding of cancer evolution and progression, as it is clear that cancer cells often manipulate their environment to alter mechanical forces in a way that favors survival and proliferation. Thus, changes in gene expression and associated patient outcomes may not necessarily be driven by genetic events such as mutation or copy number variation, which alter the activity of prosurvival and proliferative pathways, but instead may be due to changes in mechanical forces experienced by tumor cells.

## Methods

### Experimental methods

The experimental protocols for the used data sets are described in detail in the associated publications. Expression profiling in Grigoriadis et al. (2012) and image profiling in Sero et al. (2015) were performed using the same batches of cell lines and under similar culture conditions. For imaging experiments, 1000 cells per well were seeded in 384-well plates and cultured for three days. Different cell lines were monitored for cell crowding. Cells were stained with DAPI, DHE (Invitrogen), and anti-RELA/NF-κB antibody (Abcam).

### Data analysis

All analyses were performed using MatLab (http://www.mathworks.com/) unless stated otherwise.

### Identifying shape-correlated genes

The threshold of genes with low variability across BCLs was identified by plotting the distribution of SD values for all genes across BCLs. A bimodal distribution was observed where a cut-off of 0.3 separates genes with low variability versus genes with high variability across BCLs (Supplemental Fig. S4A). We measured the Spearman correlation between each shape feature against the expression of each gene. The cut-off for significant correlation is 0.7 (*P*-value < 0.0012, FDR < 23% as estimated by “mafdr” MatLab function).

### Selected TFs

The TFs that were included in the shape-gene network are: KLF4, MYC, RELA/NF-κB, SOX2, SMAD2, SMAD3, SNAI1, SNAI2, TWIST1, ZEB1 and ZEB2, and YAP1.

### Building a shape-gene interaction network

STRING interactions (Jensen et al. 2009) between shape-correlated genes and selected TFs with a combined score > 0.4 based on Neighborhood, Gene Fusion, Co-occurrence, Co-expression, Experiments, and curated Databases were downloaded (Oct. 2014). Cytoscape 2.8 (Shannon et al. 2003) was used to visualize the gene–gene and gene–feature interactions.

### Network analysis

Network node attributes were calculated using Cytoscape 2.8. Degree, closeness, and stress attributes were exported from Cytoscape and visualized in MatLab 2015a.

### Enrichment analysis

Enrichment analyses were performed using GSEA analysis provided by the Molecular Signature Database (Subramanian et al. 2005) and DAVID (Huang et al. 2009).

### Building a SMAD3-NF-κB subnetwork

Optimal and suboptimal paths between each phenotypic feature and SMAD3 or RELA were determined using the BiNom Cytoscape plugin (Bonnet et al. 2013). Indirect paths that involve other phenotypic features or other selected TFs were excluded. The interactions between the remaining genes in the path analysis were extracted to build the SMAD3-NF-κB subnetwork.

### LINCS RNAi data analysis

To determine the effect of depleting nodes in the SMAD3-NF-κB subnetwork on SMAD3 and RELA activities, the Broad Institute's LINCS RNAi genomic data set, that describes the expression of 3287 genes in the MCF7 cell line following 96 h of RNAi treatment (Duan et al. 2014), was used. Probes targeting the same gene were consolidated using GSEA software. The knockdown was only considered valid if they reduced the level of the gene to a *z*-score value < −0.4. If more than one knockdown for one gene is available, then the average profile for valid probes is used for further computation.

To determine the interaction directionality in the SMAD3-NF-κB subnetwork, we *z*-scored gene expression values across all knockdowns. Then, we defined TF targets as the genes whose expression is significantly changed after TF knockdown (absolute *z*-score > 1.5). Then, we considered the three following scenarios:

1. The protein was considered to regulate TF expression if its knockdown significantly changes the expression of that TF (absolute *z*-score >1.5).
2. The protein was considered to regulate a TF activation if
  - the protein knockdown significantly changes the expression of at least 10% of the TF target genes, and
  - the overlap between the proteins' targets and TF targets is statistically significant using hypergeometric probability (*P* < 0.05).
3. A TF was considered to regulate a gene if the TF knockdown significantly changes the expression of that gene (absolute *z*-score >1.5).

We also inferred the directionality, when possible, for existing STRING interactions in the subnetwork if the knockdown of one of the interactors significantly changes the expression of another (indicated by red arrows in Fig. 4).

For proteins that significantly change the activation of a TF, we further determined whether they activate or inhibit the TF. If the depletion of the protein affects the expression of the TF targets in a similar way as TF knockdown, then we infer that the protein activates that TF, while if the depletion of the protein has the opposite effect on the expression of the TF targets compared to knockdown of the TF, then we infer that the protein inhibits that TF.

### Analysis of the expression of SMAD3-NF-κB subnetwork's genes in BCLs

All gene profiles were *z*-scored across 18 BCLs. The profiles of genes with more than one probe were averaged. Hierarchical clustering with Euclidean distance and “complete linkage” was used to cluster BCLs and gene profiles.

### Derivation of the basal and luminal networks

The average expression profiles of luminal and basal clusters were obtained by averaging the *z*-scored expression of the genes in the SMAD3-NF-κB subnetwork for the cell lines in the luminal or basal clusters (Fig. 5B). Average profiles that have a >0.5 *z*-score difference between basal and luminal clusters were considered to be differentially expressed and included in the network. Nondifferentially expressed genes were eliminated from the network if their average expression < 7.0 as estimated based on the distribution of the expression of all genes in the data set (Supplemental Fig. S4B). Visualization was performed using Cytoscape 2.8.

### Derivation of morphological metagenes

We fitted a multilinear regression model to estimate the average or SD of a morphological feature from the expression of a selected subset of genes that correlate with that feature. The genes that best predict the feature were selected using forward sequential feature selection in MatLab where at least four genes were selected. The criterion of the model fitness is the sum of the residuals of the regression model. Genes were added to the model as long as R^2^< 0.9, and the number of selected genes ≤ 10. For the random metagene, we generated a random variable of size 18. After that, 20 genes were randomly drawn from the original subset of genes (11,314 genes). Then, the same approach for deriving the morphological metagenes was used to generate the random metagene.

### Survival analysis

Survival analyses were performed using R 3.2.0 (R Core Team 2008). Morphological metagenes were binarized based on the cut-off that best predicts patient survival based on the discovery cohort. The cut-offs were defined as {0.2, 0.3, 0.4, 0.5, 0.6, 0.7, 0.8} quantiles. The Kaplan–Meier method was used to fit survival curves. Cox proportional hazards models were used to perform univariate and multivariate analyses where the Wald test was used to measure significance. In Cox multivariate analyses, the lymph node variable was set to 1 if cancer cells have spread to at least one lymph node, and 0 otherwise. The tumor size variable was set to 1 if the tumor size > 2 cm and 0 otherwise.

## Supplementary Material

Supplemental Material
