# Supplementary material for: Identification of clinically predictive metagenes that encode components of a network coupling cell shape to transcription by image-omics
Source: Genome Res. 2017 Feb;27(2):196–207. doi: 10.1101/gr.202028.115 (PMC5287226; doi:10.1101/gr.202028.115)
Supplement: Supplemental Material [file supp_27_2_196__index.html]

Identification of clinically predictive metagenes that encode components of a network coupling cell shape to transcription by image-omics — Identification of clinically predictive metagenes that encode components of a network coupling cell shape to transcription by image-omics — Supplemental Material 

# Identification of clinically predictive metagenes that encode components of a network coupling cell shape to transcription by image-omics

## Supplemental Material

- Supplemental\_Fig\_S1-S4.docx
- Supplemental\_Table\_S1-S7.docx
- Supplemental\_Table\_S8-S11.docx
