# Supplementary material for: Identification of clinically predictive metagenes that encode components of a network coupling cell shape to transcription by image-omics
Source: Genome Res. 2017 Feb;27(2):196–207. doi: 10.1101/gr.202028.115 (PMC5287226; doi:10.1101/gr.202028.115)
Supplement: Supplemental Material [file supp_gr.202028.115_Supplemental_Fig_S1-S4.docx]

**Supplemental Figures**

**Supplemental Figure S1: Example correlation plots between morphological features (mean) and genes.** Morphological features are scaled between 0 and 1. Each dot represents a BCL. Red: luminal, green: basal A, blue: basal B.

**Supplemental Figure S2: Example correlation plots between morphological features (std) and genes.** Morphological features (std) are scaled between 0 and 1. Each dot represents a BCL. Red: luminal, green: basal A, blue: basal B.

**Supplemental Figure S3: Phenotype-genotype network analysis** (A-B) Optimal and sub-optimal paths from cell width/length feature to (A) SMAD3 and (B) RELA. Nodes in yellow indicate TFs that we included for investigation and are excluded from path analysis results. (C) Schematic of two ways a shape-correlated gene may affect a TF (using RELA as an example).


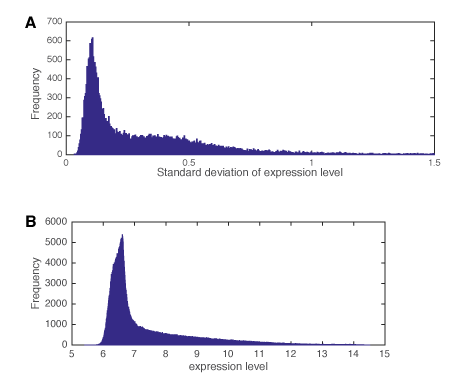


**Supplemental Figure S4: Distribution of gene expression statistics.** (A) The standard deviation and (B) average expression of all genes across 18 BCLs.
