# Supplementary material for: Identification of clinically predictive metagenes that encode components of a network coupling cell shape to transcription by image-omics
Source: Genome Res. 2017 Feb;27(2):196–207. doi: 10.1101/gr.202028.115 (PMC5287226; doi:10.1101/gr.202028.115)
Supplement: Supplemental Material [file supp_gr.202028.115_Supplemental_Table_S1-S7.docx]

**Supplemental Tables S1-S7**

Supplemental Table S1: The genetic, molecular and tumour subtypes of breast cell lines

Data is from (Neve et al., 2006).

| **Cell Line** | **Genetic cluster** | **ER status** | **PR**  **status** | **HER2**  **status** |
| --- | --- | --- | --- | --- |
| BT474 | Luminal | + | - | + |
| CAMA1 | Luminal | + | [-] | - |
| T47D | Luminal | + | [+] | - |
| ZR75.1 | Luminal | + | [-] | - |
| MCDMB453 | Luminal | - | [-] | - |
| SKBR3 | Luminal | - | [-] | + |
| MCF7 | Luminal | + | [+] | - |
| HCC1143 | Basal A | - | [-] | - |
| HCC1954 | Basal A | - | [-] | + |
| HCC70 | Basal A | - | [-] | - |
| hs578T | Basal B | - | [-] | - |
| JIMT1 | Unclassified | - | - | + |
| MCF10A | Basal B | - | [-] | - |
| MCF12A | Basal B | - | [-] | - |
| MDAMB157 | Basal B | - | [-] | - |
| MDAMB231 | Basal B | - | [-] | - |
| SUM149 | Basal B | [-] | [-] | - |
| SUM159 | Basal B | [-] | [-] | - |

Supplemental Table S2: List of the genes that their expression values are correlated with the average or standard deviation of each morphological feature

| **Feature** | **Gene** | **Spearman Correlation Coef.** | **P-value** | **Statistic** |
| --- | --- | --- | --- | --- |
| 'Cell Area' | *'AFG3L2'* | -0.70 | 0.00158 | Average |
| 'Cell Area' | *'ATP2C1'* | 0.72 | 0.00100 | Average |
| 'Cell Area' | *'ATP6V0E2'* | -0.70 | 0.00158 | Average |
| 'Cell Area' | *'C20orf149'* | -0.72 | 0.00100 | Average |
| 'Cell Area' | *'CSGALNACT2'* | 0.72 | 0.00100 | Average |
| 'Cell Area' | *'CSNK1G3'* | 0.73 | 0.00095 | Average |
| 'Cell Area' | *'DCXR'* | -0.77 | 0.00029 | Average |
| 'Cell Area' | *'ERLIN1'* | 0.74 | 0.00067 | Average |
| 'Cell Area' | *'GIGYF2'* | -0.70 | 0.00165 | Average |
| 'Cell Area' | *'HES4'* | -0.72 | 0.00110 | Average |
| 'Cell Area' | *'IDH3G'* | -0.78 | 0.00024 | Average |
| 'Cell Area' | *'ITGB1'* | 0.70 | 0.00152 | Average |
| 'Cell Area' | *'KCTD14'* | 0.74 | 0.00063 | Average |
| 'Cell Area' | *'KIF1B'* | -0.73 | 0.00091 | Average |
| 'Cell Area' | *'NDUFB7'* | -0.73 | 0.00095 | Average |
| 'Cell Area' | *'NFE2L1'* | 0.79 | 0.00013 | Average |
| 'Cell Area' | *'PAM'* | 0.80 | 0.00010 | Average |
| 'Cell Area' | *'PELO'* | 0.71 | 0.00139 | Average |
| 'Cell Area' | *'PLXNA1'* | 0.80 | 0.00008 | Average |
| 'Cell Area' | *'POFUT1'* | 0.71 | 0.00145 | Average |
| 'Cell Area' | *'PTK2'* | 0.79 | 0.00014 | Average |
| 'Cell Area' | *'RAD9A'* | -0.76 | 0.00033 | Average |
| 'Cell Area' | *'RAGE'* | 0.75 | 0.00054 | Average |
| 'Cell Area' | *'RP11-398K22.9'* | 0.74 | 0.00070 | Average |
| 'Cell Area' | *'SDR'* | 0.75 | 0.00054 | Average |
| 'Cell Area' | *'SYT12'* | -0.70 | 0.00152 | Average |
| 'Cell Area' | *'TMEM44'* | 0.73 | 0.00095 | Average |
| 'Cell Area' | *'TUBGCP4'* | -0.75 | 0.00057 | Average |
| 'Cell Area' | *'CHRAC1'* | -0.77 | 0.00029 | Std |
| 'Cell Area' | *'CHST15'* | -0.74 | 0.00067 | Std |
| 'Cell Area' | *'LARP5'* | 0.74 | 0.00070 | Std |
| 'Cell Area' | *'LRRC34'* | -0.73 | 0.00095 | Std |
| 'Cell Width/Length' | *'ABCA12'* | -0.73 | 0.00086 | Average |
| 'Cell Width/Length' | *'AC025257.22'* | -0.70 | 0.00158 | Average |
| 'Cell Width/Length' | *'AC108938.4'* | 0.73 | 0.00086 | Average |
| 'Cell Width/Length' | *'ANKRD36'* | 0.72 | 0.00121 | Average |
| 'Cell Width/Length' | *'ANLN'* | 0.77 | 0.00028 | Average |
| 'Cell Width/Length' | *'ATP2C1'* | 0.72 | 0.00100 | Average |
| 'Cell Width/Length' | *'ATPGD1'* | -0.79 | 0.00012 | Average |
| 'Cell Width/Length' | *'C1orf35'* | -0.75 | 0.00054 | Average |
| 'Cell Width/Length' | *'CD47'* | 0.73 | 0.00091 | Average |
| 'Cell Width/Length' | *'CHST8'* | -0.75 | 0.00057 | Average |
| 'Cell Width/Length' | *'CNTNAP3B'* | 0.74 | 0.00067 | Average |
| 'Cell Width/Length' | *'COL18A1'* | 0.74 | 0.00074 | Average |
| 'Cell Width/Length' | *'CRIM1'* | 0.73 | 0.00091 | Average |
| 'Cell Width/Length' | *'DDAH1'* | 0.75 | 0.00048 | Average |
| 'Cell Width/Length' | *'DEPDC1'* | 0.74 | 0.00067 | Average |
| 'Cell Width/Length' | *'DPY19L1'* | 0.71 | 0.00126 | Average |
| 'Cell Width/Length' | *'ENTPD2'* | -0.70 | 0.00165 | Average |
| 'Cell Width/Length' | *'FABP7'* | -0.74 | 0.00060 | Average |
| 'Cell Width/Length' | *'FIGN'* | 0.86 | 0.00000 | Average |
| 'Cell Width/Length' | *'FOXD4L2'* | -0.73 | 0.00082 | Average |
| 'Cell Width/Length' | *'FOXD4L5'* | -0.71 | 0.00139 | Average |
| 'Cell Width/Length' | *'FZD7'* | 0.74 | 0.00070 | Average |
| 'Cell Width/Length' | *'GAK'* | -0.73 | 0.00095 | Average |
| 'Cell Width/Length' | *'GLT25D2'* | 0.77 | 0.00028 | Average |
| 'Cell Width/Length' | *'GRHL1'* | -0.73 | 0.00086 | Average |
| 'Cell Width/Length' | *'GYPC'* | 0.81 | 0.00007 | Average |
| 'Cell Width/Length' | *'HDAC9'* | 0.70 | 0.00165 | Average |
| 'Cell Width/Length' | *'HECW2'* | 0.72 | 0.00110 | Average |
| 'Cell Width/Length' | *'HNT'* | 0.72 | 0.00105 | Average |
| 'Cell Width/Length' | *'HSD17B10'* | -0.83 | 0.00001 | Average |
| 'Cell Width/Length' | *'ICAM3'* | -0.70 | 0.00158 | Average |
| 'Cell Width/Length' | *'INHA'* | -0.81 | 0.00006 | Average |
| 'Cell Width/Length' | *'ITPKB'* | -0.70 | 0.00158 | Average |
| 'Cell Width/Length' | *'JMJD4'* | -0.79 | 0.00016 | Average |
| 'Cell Width/Length' | *'KIAA1984'* | -0.72 | 0.00100 | Average |
| 'Cell Width/Length' | *'LEPREL1'* | 0.74 | 0.00070 | Average |
| 'Cell Width/Length' | *'LIFR'* | 0.81 | 0.00006 | Average |
| 'Cell Width/Length' | *'LYPD3'* | -0.74 | 0.00067 | Average |
| 'Cell Width/Length' | *'MAP1B'* | 0.72 | 0.00121 | Average |
| 'Cell Width/Length' | *'MYLK'* | 0.70 | 0.00152 | Average |
| 'Cell Width/Length' | *'NBPF1'* | -0.77 | 0.00031 | Average |
| 'Cell Width/Length' | *'NRP1'* | 0.72 | 0.00121 | Average |
| 'Cell Width/Length' | *'NUP155'* | 0.71 | 0.00126 | Average |
| 'Cell Width/Length' | *'OLFML2A'* | 0.78 | 0.00020 | Average |
| 'Cell Width/Length' | *'OSBPL2'* | -0.72 | 0.00105 | Average |
| 'Cell Width/Length' | *'PHACTR2'* | 0.71 | 0.00145 | Average |
| 'Cell Width/Length' | *'PPBP'* | -0.71 | 0.00126 | Average |
| 'Cell Width/Length' | *'PPP2R3A'* | 0.70 | 0.00152 | Average |
| 'Cell Width/Length' | *'PVRL1'* | -0.73 | 0.00078 | Average |
| 'Cell Width/Length' | *'QKI'* | 0.76 | 0.00033 | Average |
| 'Cell Width/Length' | *'QKI'* | 0.80 | 0.00009 | Average |
| 'Cell Width/Length' | *'RCN3'* | 0.80 | 0.00009 | Average |
| 'Cell Width/Length' | *'ROR1'* | 0.76 | 0.00040 | Average |
| 'Cell Width/Length' | *'RP1-286D6.2'* | -0.73 | 0.00086 | Average |
| 'Cell Width/Length' | *'RP11-4L24.2'* | -0.79 | 0.00016 | Average |
| 'Cell Width/Length' | *'SEC11L3'* | -0.74 | 0.00063 | Average |
| 'Cell Width/Length' | *'SLC27A6'* | -0.76 | 0.00040 | Average |
| 'Cell Width/Length' | *'SLC46A2'* | -0.71 | 0.00139 | Average |
| 'Cell Width/Length' | *'SMAD3'* | 0.70 | 0.00165 | Average |
| 'Cell Width/Length' | *'SPANXB2'* | 0.75 | 0.00054 | Average |
| 'Cell Width/Length' | *'TLCD1'* | -0.73 | 0.00078 | Average |
| 'Cell Width/Length' | *'TMEM144'* | -0.73 | 0.00082 | Average |
| 'Cell Width/Length' | *'TMEM205'* | -0.71 | 0.00126 | Average |
| 'Cell Width/Length' | *'TPCN2'* | -0.75 | 0.00048 | Average |
| 'Cell Width/Length' | *'TRIO'* | 0.75 | 0.00045 | Average |
| 'Cell Width/Length' | *'UACA'* | 0.74 | 0.00070 | Average |
| 'Cell Width/Length' | *'UFC1'* | -0.72 | 0.00115 | Average |
| 'Cell Width/Length' | *'WDR36'* | 0.71 | 0.00132 | Average |
| 'Cell Width/Length' | *'ABCC12'* | -0.71 | 0.00145 | Std |
| 'Cell Width/Length' | *'AC002454.1'* | 0.71 | 0.00132 | Std |
| 'Cell Width/Length' | *'AC007362.4'* | 0.79 | 0.00014 | Std |
| 'Cell Width/Length' | *'ADAMTSL1'* | 0.71 | 0.00139 | Std |
| 'Cell Width/Length' | *'ADC'* | 0.74 | 0.00063 | Std |
| 'Cell Width/Length' | *'AFAP1L1'* | 0.81 | 0.00005 | Std |
| 'Cell Width/Length' | *'ARFGEF2'* | -0.70 | 0.00152 | Std |
| 'Cell Width/Length' | *'ATMIN'* | -0.81 | 0.00006 | Std |
| 'Cell Width/Length' | *'ATP6V1G1'* | -0.74 | 0.00067 | Std |
| 'Cell Width/Length' | *'BLNK'* | -0.74 | 0.00067 | Std |
| 'Cell Width/Length' | *'C10orf140'* | 0.73 | 0.00078 | Std |
| 'Cell Width/Length' | *'C16orf76'* | -0.71 | 0.00145 | Std |
| 'Cell Width/Length' | *'C17orf28'* | -0.71 | 0.00145 | Std |
| 'Cell Width/Length' | *'C9orf163'* | -0.79 | 0.00014 | Std |
| 'Cell Width/Length' | *'CCDC88A'* | 0.76 | 0.00033 | Std |
| 'Cell Width/Length' | *'COL5A1'* | 0.85 | 0.00000 | Std |
| 'Cell Width/Length' | *'CPAMD8'* | -0.72 | 0.00110 | Std |
| 'Cell Width/Length' | *'CPOX'* | 0.72 | 0.00110 | Std |
| 'Cell Width/Length' | *'CREBZF'* | -0.72 | 0.00105 | Std |
| 'Cell Width/Length' | *'DFFA'* | 0.74 | 0.00067 | Std |
| 'Cell Width/Length' | *'DULLARD'* | 0.72 | 0.00110 | Std |
| 'Cell Width/Length' | *'EMP3'* | 0.70 | 0.00152 | Std |
| 'Cell Width/Length' | *'EPN3'* | -0.75 | 0.00048 | Std |
| 'Cell Width/Length' | *'ETS1'* | 0.71 | 0.00139 | Std |
| 'Cell Width/Length' | *'FAM174B'* | -0.75 | 0.00048 | Std |
| 'Cell Width/Length' | *'FBXO17'* | 0.73 | 0.00082 | Std |
| 'Cell Width/Length' | *'FTS'* | -0.76 | 0.00038 | Std |
| 'Cell Width/Length' | *'GRHL2'* | -0.78 | 0.00021 | Std |
| 'Cell Width/Length' | *'H2AFJ'* | -0.73 | 0.00086 | Std |
| 'Cell Width/Length' | *'H2AFJ'* | -0.72 | 0.00100 | Std |
| 'Cell Width/Length' | *'HBEGF'* | 0.72 | 0.00110 | Std |
| 'Cell Width/Length' | *'HIST2H4B'* | -0.71 | 0.00126 | Std |
| 'Cell Width/Length' | *'HLA-H'* | 0.74 | 0.00063 | Std |
| 'Cell Width/Length' | *'KIFC3'* | 0.74 | 0.00067 | Std |
| 'Cell Width/Length' | *'LAPTM5'* | 0.75 | 0.00045 | Std |
| 'Cell Width/Length' | *'LAYN'* | 0.71 | 0.00132 | Std |
| 'Cell Width/Length' | *'MANSC1'* | -0.70 | 0.00152 | Std |
| 'Cell Width/Length' | *'MAP4K2'* | -0.70 | 0.00165 | Std |
| 'Cell Width/Length' | *'MARVELD3'* | -0.72 | 0.00121 | Std |
| 'Cell Width/Length' | *'MARVELD3'* | -0.73 | 0.00078 | Std |
| 'Cell Width/Length' | *'MICAL1'* | 0.78 | 0.00022 | Std |
| 'Cell Width/Length' | *'MRPL49'* | -0.82 | 0.00004 | Std |
| 'Cell Width/Length' | *'NACAD'* | 0.74 | 0.00070 | Std |
| 'Cell Width/Length' | *'NAV3'* | 0.74 | 0.00070 | Std |
| 'Cell Width/Length' | *'NHLRC3'* | -0.77 | 0.00031 | Std |
| 'Cell Width/Length' | *'NXN'* | 0.71 | 0.00139 | Std |
| 'Cell Width/Length' | *'ODC1'* | 0.74 | 0.00074 | Std |
| 'Cell Width/Length' | *'PHTF1'* | 0.73 | 0.00095 | Std |
| 'Cell Width/Length' | *'PMP22'* | 0.73 | 0.00091 | Std |
| 'Cell Width/Length' | *'PTX3'* | 0.79 | 0.00016 | Std |
| 'Cell Width/Length' | *'RAB11A'* | -0.79 | 0.00016 | Std |
| 'Cell Width/Length' | *'RANBP10'* | -0.76 | 0.00036 | Std |
| 'Cell Width/Length' | *'RCN3'* | 0.72 | 0.00121 | Std |
| 'Cell Width/Length' | *'S100A4'* | 0.72 | 0.00110 | Std |
| 'Cell Width/Length' | *'SCHIP1'* | 0.70 | 0.00165 | Std |
| 'Cell Width/Length' | *'SCYL2'* | -0.79 | 0.00016 | Std |
| 'Cell Width/Length' | *'SCYL3'* | -0.72 | 0.00115 | Std |
| 'Cell Width/Length' | *'SCYL3'* | -0.74 | 0.00067 | Std |
| 'Cell Width/Length' | *'SH2D5'* | 0.77 | 0.00029 | Std |
| 'Cell Width/Length' | *'SLC9A3R1'* | -0.71 | 0.00145 | Std |
| 'Cell Width/Length' | *'SPTBN1'* | 0.71 | 0.00139 | Std |
| 'Cell Width/Length' | *'SRI'* | 0.72 | 0.00105 | Std |
| 'Cell Width/Length' | *'STX19'* | -0.74 | 0.00070 | Std |
| 'Cell Width/Length' | *'TMEM79'* | -0.75 | 0.00057 | Std |
| 'Cell Width/Length' | *'TRAPPC3'* | 0.70 | 0.00152 | Std |
| 'Cell Width/Length' | *'UTP14C'* | -0.70 | 0.00158 | Std |
| 'Cell Width/Length' | *'ZNF263'* | -0.70 | 0.00158 | Std |
| 'Centers Distance' | *'AC006033.2-4'* | 0.71 | 0.00145 | Average |
| 'Centers Distance' | *'AC011511.11'* | -0.75 | 0.00051 | Average |
| 'Centers Distance' | *'ADM'* | 0.75 | 0.00048 | Average |
| 'Centers Distance' | *'ATG10'* | 0.73 | 0.00082 | Average |
| 'Centers Distance' | *'BX248398.1'* | 0.70 | 0.00165 | Average |
| 'Centers Distance' | *'C6orf64'* | -0.71 | 0.00139 | Average |
| 'Centers Distance' | *'CES8'* | -0.78 | 0.00018 | Average |
| 'Centers Distance' | *'CYTL1'* | 0.73 | 0.00086 | Average |
| 'Centers Distance' | *'DHX34'* | -0.75 | 0.00057 | Average |
| 'Centers Distance' | *'DMD'* | 0.75 | 0.00045 | Average |
| 'Centers Distance' | *'FAM173A'* | -0.73 | 0.00086 | Average |
| 'Centers Distance' | *'FRMD3'* | 0.74 | 0.00067 | Average |
| 'Centers Distance' | *'KIAA1467'* | -0.71 | 0.00145 | Average |
| 'Centers Distance' | *'KIAA1644'* | 0.73 | 0.00082 | Average |
| 'Centers Distance' | *'LY6G5C'* | -0.70 | 0.00165 | Average |
| 'Centers Distance' | *'N4BP2L2'* | -0.70 | 0.00152 | Average |
| 'Centers Distance' | *'NCRNA00176'* | -0.74 | 0.00074 | Average |
| 'Centers Distance' | *'PCBD1'* | -0.71 | 0.00126 | Average |
| 'Centers Distance' | *'PVALB'* | -0.79 | 0.00014 | Average |
| 'Centers Distance' | *'RNASET2'* | -0.71 | 0.00132 | Average |
| 'Centers Distance' | *'RNF145'* | 0.76 | 0.00036 | Average |
| 'Centers Distance' | *'RP11-398K22.9'* | 0.71 | 0.00145 | Average |
| 'Centers Distance' | *'TNS3'* | 0.71 | 0.00126 | Average |
| 'Centers Distance' | *'UBE2G2'* | 0.75 | 0.00057 | Average |
| 'Centers Distance' | *'USP21'* | -0.72 | 0.00121 | Average |
| 'Centers Distance' | *'VRK3'* | -0.71 | 0.00132 | Average |
| 'Centers Distance' | *'VSIG2'* | -0.74 | 0.00074 | Average |
| 'Centers Distance' | *'AC011511.11'* | -0.74 | 0.00060 | Std |
| 'Centers Distance' | *'FAM183A'* | 0.75 | 0.00054 | Std |
| 'Centers Distance' | *'JPH3'* | -0.72 | 0.00121 | Std |
| 'Centers Distance' | *'LRRC24'* | -0.71 | 0.00132 | Std |
| 'Centers Distance' | *'RAB9B'* | -0.72 | 0.00115 | Std |
| 'Centers Distance' | *'UBE2G2'* | 0.76 | 0.00036 | Std |
| 'Neighbor Fraction' | *'AARS'* | 0.76 | 0.00040 | Average |
| 'Neighbor Fraction' | *'AGTPBP1'* | -0.70 | 0.00158 | Average |
| 'Neighbor Fraction' | *'ARL4A'* | -0.74 | 0.00074 | Average |
| 'Neighbor Fraction' | *'C20orf29'* | -0.72 | 0.00105 | Average |
| 'Neighbor Fraction' | *'CDH11'* | -0.73 | 0.00086 | Average |
| 'Neighbor Fraction' | *'HAS2'* | -0.87 | 0.00000 | Average |
| 'Neighbor Fraction' | *'HSD17B7P2'* | 0.71 | 0.00139 | Average |
| 'Neighbor Fraction' | *'KLHL13'* | 0.74 | 0.00063 | Average |
| 'Neighbor Fraction' | *'NUBPL'* | 0.73 | 0.00082 | Average |
| 'Neighbor Fraction' | *'PDCD6'* | 0.74 | 0.00063 | Average |
| 'Neighbor Fraction' | *'PDE7B'* | -0.86 | 0.00000 | Average |
| 'Neighbor Fraction' | *'PDLIM7'* | -0.81 | 0.00006 | Average |
| 'Neighbor Fraction' | *'RANGRF'* | -0.74 | 0.00063 | Average |
| 'Neighbor Fraction' | *'RAX2'* | -0.73 | 0.00095 | Average |
| 'Neighbor Fraction' | *'RGS4'* | -0.71 | 0.00139 | Average |
| 'Neighbor Fraction' | *'RHOT1'* | 0.77 | 0.00026 | Average |
| 'Neighbor Fraction' | *'RHOT1'* | 0.70 | 0.00158 | Average |
| 'Neighbor Fraction' | *'RNASEK'* | -0.71 | 0.00132 | Average |
| 'Neighbor Fraction' | *'TEX261'* | -0.75 | 0.00051 | Average |
| 'Neighbor Fraction' | *'TRAPPC1'* | -0.71 | 0.00126 | Average |
| 'Neighbor Fraction' | *'ZNF280B'* | 0.72 | 0.00115 | Average |
| 'Neighbor Fraction' | *'AC008268.3'* | 0.74 | 0.00070 | Std |
| 'Neighbor Fraction' | *'ANKRD37'* | -0.71 | 0.00126 | Std |
| 'Neighbor Fraction' | *'BMP4'* | -0.71 | 0.00145 | Std |
| 'Neighbor Fraction' | *'BTBD11'* | 0.80 | 0.00008 | Std |
| 'Neighbor Fraction' | *'C11orf87'* | -0.72 | 0.00105 | Std |
| 'Neighbor Fraction' | *'C2orf49'* | 0.75 | 0.00051 | Std |
| 'Neighbor Fraction' | *'C6orf115'* | 0.72 | 0.00100 | Std |
| 'Neighbor Fraction' | *'CD151'* | -0.73 | 0.00078 | Std |
| 'Neighbor Fraction' | *'CHMP4C'* | 0.71 | 0.00139 | Std |
| 'Neighbor Fraction' | *'CNPY4'* | -0.76 | 0.00036 | Std |
| 'Neighbor Fraction' | *'DEAF1'* | -0.82 | 0.00004 | Std |
| 'Neighbor Fraction' | *'GSTM1'* | -0.73 | 0.00082 | Std |
| 'Neighbor Fraction' | *'GSTM4'* | -0.72 | 0.00105 | Std |
| 'Neighbor Fraction' | *'KLK7'* | 0.76 | 0.00036 | Std |
| 'Neighbor Fraction' | *'KLK8'* | 0.75 | 0.00054 | Std |
| 'Neighbor Fraction' | *'MSL3'* | -0.76 | 0.00043 | Std |
| 'Neighbor Fraction' | *'PADI4'* | 0.72 | 0.00100 | Std |
| 'Neighbor Fraction' | *'PARN'* | 0.71 | 0.00139 | Std |
| 'Neighbor Fraction' | *'RP11-20F24.4'* | -0.70 | 0.00158 | Std |
| 'Neighbor Fraction' | *'SFRS14'* | -0.80 | 0.00011 | Std |
| 'Neighbor Fraction' | *'SPRR1B'* | 0.78 | 0.00024 | Std |
| 'Neighbor Fraction' | *'TMEM40'* | 0.70 | 0.00152 | Std |
| 'Neighbor Fraction' | *'WNT7A'* | 0.73 | 0.00082 | Std |
| 'Neighbor Fraction' | *'ZNF330'* | -0.74 | 0.00074 | Std |
| 'Nuclear Area' | *'AC087742.9'* | 0.72 | 0.00105 | Average |
| 'Nuclear Area' | *'C2orf82'* | -0.74 | 0.00074 | Average |
| 'Nuclear Area' | *'OTUD6B'* | 0.78 | 0.00022 | Average |
| 'Nuclear Area' | *'SCCPDH'* | -0.74 | 0.00067 | Average |
| 'Nuclear Area' | *'TATDN1'* | 0.70 | 0.00165 | Average |
| 'Nuclear Area' | *'UBE2G2'* | 0.74 | 0.00063 | Average |
| 'Nuclear Area' | *'UTP23'* | 0.79 | 0.00017 | Average |
| 'Nuclear Area' | *'ANKRD50'* | -0.71 | 0.00132 | Std |
| 'Nuclear Area' | *'AP005212.2-2'* | 0.76 | 0.00033 | Std |
| 'Nuclear Area' | *'ARG2'* | -0.79 | 0.00017 | Std |
| 'Nuclear Area' | *'C9orf82'* | -0.72 | 0.00110 | Std |
| 'Nuclear Area' | *'CITED4'* | 0.70 | 0.00158 | Std |
| 'Nuclear Area' | *'CNTN1'* | 0.76 | 0.00040 | Std |
| 'Nuclear Area' | *'FAM25A'* | 0.74 | 0.00060 | Std |
| 'Nuclear Area' | *'IDH3B'* | 0.71 | 0.00126 | Std |
| 'Nuclear Area' | *'MFAP3L'* | 0.74 | 0.00074 | Std |
| 'Nuclear Area' | *'NSFL1C'* | 0.75 | 0.00051 | Std |
| 'Nuclear Area' | *'OLR1'* | 0.70 | 0.00158 | Std |
| 'Nuclear Roundness' | *'AC002351.2'* | 0.76 | 0.00038 | Average |
| 'Nuclear Roundness' | *'AKAP8L'* | -0.73 | 0.00086 | Average |
| 'Nuclear Roundness' | *'ALDH1L2'* | 0.80 | 0.00010 | Average |
| 'Nuclear Roundness' | *'ANAPC13'* | 0.77 | 0.00029 | Average |
| 'Nuclear Roundness' | *'C19orf63'* | -0.75 | 0.00054 | Average |
| 'Nuclear Roundness' | *'C1orf93'* | -0.73 | 0.00095 | Average |
| 'Nuclear Roundness' | *'C9orf6'* | 0.83 | 0.00000 | Average |
| 'Nuclear Roundness' | *'CCDC53'* | 0.70 | 0.00165 | Average |
| 'Nuclear Roundness' | *'CRLF1'* | 0.73 | 0.00091 | Average |
| 'Nuclear Roundness' | *'CTA-221G9.5'* | -0.72 | 0.00115 | Average |
| 'Nuclear Roundness' | *'EDN2'* | -0.73 | 0.00095 | Average |
| 'Nuclear Roundness' | *'EIF2A'* | 0.70 | 0.00152 | Average |
| 'Nuclear Roundness' | *'FAM108A4'* | -0.72 | 0.00115 | Average |
| 'Nuclear Roundness' | *'FAM5C'* | -0.71 | 0.00126 | Average |
| 'Nuclear Roundness' | *'FNDC5'* | 0.80 | 0.00008 | Average |
| 'Nuclear Roundness' | *'GARNL3'* | -0.70 | 0.00152 | Average |
| 'Nuclear Roundness' | *'GRWD1'* | -0.75 | 0.00057 | Average |
| 'Nuclear Roundness' | *'IPO8'* | -0.77 | 0.00026 | Average |
| 'Nuclear Roundness' | *'NDUFA9'* | -0.72 | 0.00115 | Average |
| 'Nuclear Roundness' | *'PSME2'* | -0.72 | 0.00115 | Average |
| 'Nuclear Roundness' | *'PUSL1'* | -0.71 | 0.00126 | Average |
| 'Nuclear Roundness' | *'SYT12'* | -0.73 | 0.00078 | Average |
| 'Nuclear Roundness' | *'TOX3'* | -0.82 | 0.00004 | Average |
| 'Nuclear Roundness' | *'TRMU'* | -0.72 | 0.00115 | Average |
| 'Nuclear Roundness' | *'UBE2V2'* | 0.72 | 0.00110 | Average |
| 'Nuclear Roundness' | *'ANKRD24'* | -0.73 | 0.00082 | Std |
| 'Nuclear Roundness' | *'AP3S1'* | -0.81 | 0.00007 | Std |
| 'Nuclear Roundness' | *'GLT8D1'* | -0.71 | 0.00139 | Std |
| 'Nuclear Roundness' | *'IDH3B'* | 0.85 | 0.00000 | Std |
| 'Nuclear Roundness' | *'ILVBL'* | 0.71 | 0.00126 | Std |
| 'Nuclear Roundness' | *'IMMP1L'* | 0.70 | 0.00158 | Std |
| 'Nuclear Roundness' | *'ISCU'* | -0.73 | 0.00095 | Std |
| 'Nuclear Roundness' | *'KIAA1128'* | -0.82 | 0.00002 | Std |
| 'Nuclear Roundness' | *'MAP2K6'* | 0.71 | 0.00132 | Std |
| 'Nuclear Roundness' | *'MRPL12'* | 0.74 | 0.00067 | Std |
| 'Nuclear Roundness' | *'MRPS18A'* | 0.77 | 0.00026 | Std |
| 'Nuclear Roundness' | *'MRPS26'* | 0.72 | 0.00110 | Std |
| 'Nuclear Roundness' | *'NARF'* | 0.76 | 0.00040 | Std |
| 'Nuclear Roundness' | *'RAC3'* | 0.72 | 0.00110 | Std |
| 'Nuclear Roundness' | *'SLC41A3'* | -0.71 | 0.00145 | Std |
| 'Nuclear Roundness' | *'TMEM70'* | 0.81 | 0.00006 | Std |
| 'Nuclear Roundness' | *'XAGE1'* | -0.72 | 0.00110 | Std |
| 'Nuclear Roundness' | *'ZNF25'* | -0.78 | 0.00021 | Std |
| 'Nuclear Width/Length' | *'COX7A2L'* | -0.73 | 0.00095 | Average |
| 'Nuclear Width/Length' | *'CRIPT'* | -0.70 | 0.00165 | Average |
| 'Nuclear Width/Length' | *'CRLF1'* | -0.72 | 0.00110 | Average |
| 'Nuclear Width/Length' | *'DDX59'* | -0.72 | 0.00110 | Average |
| 'Nuclear Width/Length' | *'ENO3'* | -0.71 | 0.00139 | Average |
| 'Nuclear Width/Length' | *'FAM108A4'* | 0.72 | 0.00121 | Average |
| 'Nuclear Width/Length' | *'IFNAR2'* | -0.75 | 0.00057 | Average |
| 'Nuclear Width/Length' | *'LMNA'* | 0.71 | 0.00145 | Average |
| 'Nuclear Width/Length' | *'MED25'* | 0.72 | 0.00121 | Average |
| 'Nuclear Width/Length' | *'MEX3D'* | 0.73 | 0.00095 | Average |
| 'Nuclear Width/Length' | *'NUP188'* | 0.70 | 0.00152 | Average |
| 'Nuclear Width/Length' | *'PAICS'* | 0.72 | 0.00100 | Average |
| 'Nuclear Width/Length' | *'PCNX'* | -0.71 | 0.00126 | Average |
| 'Nuclear Width/Length' | *'PSMB5'* | 0.75 | 0.00051 | Average |
| 'Nuclear Width/Length' | *'AC008073.5'* | -0.72 | 0.00115 | Std |
| 'Nuclear Width/Length' | *'AC008500.7'* | 0.71 | 0.00139 | Std |
| 'Nuclear Width/Length' | *'BET1'* | -0.70 | 0.00158 | Std |
| 'Nuclear Width/Length' | *'C15orf61'* | -0.75 | 0.00054 | Std |
| 'Nuclear Width/Length' | *'C19orf63'* | 0.73 | 0.00082 | Std |
| 'Nuclear Width/Length' | *'C1orf63'* | -0.71 | 0.00126 | Std |
| 'Nuclear Width/Length' | *'CLCN6'* | -0.73 | 0.00082 | Std |
| 'Nuclear Width/Length' | *'COX7A2L'* | -0.82 | 0.00002 | Std |
| 'Nuclear Width/Length' | *'CTTN'* | 0.70 | 0.00152 | Std |
| 'Nuclear Width/Length' | *'DHX58'* | 0.74 | 0.00070 | Std |
| 'Nuclear Width/Length' | *'DOM3Z'* | -0.71 | 0.00126 | Std |
| 'Nuclear Width/Length' | *'DPYSL4'* | -0.71 | 0.00126 | Std |
| 'Nuclear Width/Length' | *'FAM108A4'* | 0.76 | 0.00036 | Std |
| 'Nuclear Width/Length' | *'FAM111B'* | 0.73 | 0.00082 | Std |
| 'Nuclear Width/Length' | *'FNDC5'* | -0.71 | 0.00132 | Std |
| 'Nuclear Width/Length' | *'GNPAT'* | -0.73 | 0.00091 | Std |
| 'Nuclear Width/Length' | *'MED25'* | 0.82 | 0.00004 | Std |
| 'Nuclear Width/Length' | *'MED8'* | -0.75 | 0.00045 | Std |
| 'Nuclear Width/Length' | *'NDUFS6'* | 0.73 | 0.00095 | Std |
| 'Nuclear Width/Length' | *'NUP210L'* | 0.78 | 0.00018 | Std |
| 'Nuclear Width/Length' | *'ORMDL1'* | -0.70 | 0.00165 | Std |
| 'Nuclear Width/Length' | *'PCNX'* | -0.74 | 0.00070 | Std |
| 'Nuclear Width/Length' | *'PEX3'* | -0.76 | 0.00040 | Std |
| 'Nuclear Width/Length' | *'PMF1'* | 0.70 | 0.00158 | Std |
| 'Nuclear Width/Length' | *'PSME1'* | 0.70 | 0.00158 | Std |
| 'Nuclear Width/Length' | *'PVRL2'* | 0.76 | 0.00033 | Std |
| 'Nuclear Width/Length' | *'SLC2A8'* | 0.79 | 0.00014 | Std |
| 'Nuclear Width/Length' | *'TMEM160'* | 0.73 | 0.00082 | Std |
| 'Nuclear Width/Length' | *'TOX3'* | 0.72 | 0.00100 | Std |
| 'Nuclear Width/Length' | *'WNT7B'* | 0.73 | 0.00086 | Std |
| 'Nuclear Width/Length' | *'XPNPEP3'* | 0.70 | 0.00165 | Std |
| 'Nuclear/Cytoplasm Area' | *'AC002454.1'* | -0.71 | 0.00145 | Average |
| 'Nuclear/Cytoplasm Area' | *'AKAP12'* | -0.74 | 0.00070 | Average |
| 'Nuclear/Cytoplasm Area' | *'AR'* | 0.76 | 0.00043 | Average |
| 'Nuclear/Cytoplasm Area' | *'ARF3'* | 0.72 | 0.00121 | Average |
| 'Nuclear/Cytoplasm Area' | *'C16orf59'* | 0.73 | 0.00082 | Average |
| 'Nuclear/Cytoplasm Area' | *'C20orf30'* | -0.71 | 0.00145 | Average |
| 'Nuclear/Cytoplasm Area' | *'C9orf114'* | 0.82 | 0.00002 | Average |
| 'Nuclear/Cytoplasm Area' | *'C9orf86'* | 0.81 | 0.00005 | Average |
| 'Nuclear/Cytoplasm Area' | *'CALD1'* | -0.75 | 0.00045 | Average |
| 'Nuclear/Cytoplasm Area' | *'CALU'* | -0.77 | 0.00026 | Average |
| 'Nuclear/Cytoplasm Area' | *'CASP2'* | 0.70 | 0.00152 | Average |
| 'Nuclear/Cytoplasm Area' | *'CD68'* | -0.72 | 0.00105 | Average |
| 'Nuclear/Cytoplasm Area' | *'CNOT1'* | 0.73 | 0.00078 | Average |
| 'Nuclear/Cytoplasm Area' | *'COG4'* | 0.70 | 0.00165 | Average |
| 'Nuclear/Cytoplasm Area' | *'COL4A1'* | -0.77 | 0.00029 | Average |
| 'Nuclear/Cytoplasm Area' | *'COL4A2'* | -0.75 | 0.00057 | Average |
| 'Nuclear/Cytoplasm Area' | *'COL6A2'* | -0.82 | 0.00003 | Average |
| 'Nuclear/Cytoplasm Area' | *'CSF1R'* | -0.72 | 0.00121 | Average |
| 'Nuclear/Cytoplasm Area' | *'CTSB'* | -0.71 | 0.00126 | Average |
| 'Nuclear/Cytoplasm Area' | *'CTSL1'* | -0.71 | 0.00132 | Average |
| 'Nuclear/Cytoplasm Area' | *'DERL2'* | -0.70 | 0.00152 | Average |
| 'Nuclear/Cytoplasm Area' | *'EBP'* | 0.70 | 0.00152 | Average |
| 'Nuclear/Cytoplasm Area' | *'EFNA4'* | 0.72 | 0.00105 | Average |
| 'Nuclear/Cytoplasm Area' | *'EHBP1'* | -0.71 | 0.00132 | Average |
| 'Nuclear/Cytoplasm Area' | *'ELOVL4'* | -0.72 | 0.00110 | Average |
| 'Nuclear/Cytoplasm Area' | *'EMILIN2'* | -0.83 | 0.00001 | Average |
| 'Nuclear/Cytoplasm Area' | *'FAM173A'* | 0.74 | 0.00074 | Average |
| 'Nuclear/Cytoplasm Area' | *'FAM43B'* | -0.73 | 0.00091 | Average |
| 'Nuclear/Cytoplasm Area' | *'FUK'* | 0.75 | 0.00051 | Average |
| 'Nuclear/Cytoplasm Area' | *'GPX7'* | -0.71 | 0.00145 | Average |
| 'Nuclear/Cytoplasm Area' | *'H1FX'* | 0.75 | 0.00054 | Average |
| 'Nuclear/Cytoplasm Area' | *'HAPLN3'* | -0.77 | 0.00029 | Average |
| 'Nuclear/Cytoplasm Area' | *'HMGA2'* | -0.71 | 0.00139 | Average |
| 'Nuclear/Cytoplasm Area' | *'HSD17B7P2'* | 0.76 | 0.00033 | Average |
| 'Nuclear/Cytoplasm Area' | *'IFNAR2'* | -0.77 | 0.00026 | Average |
| 'Nuclear/Cytoplasm Area' | *'IGFBP7'* | -0.71 | 0.00132 | Average |
| 'Nuclear/Cytoplasm Area' | *'ITGA1'* | -0.70 | 0.00158 | Average |
| 'Nuclear/Cytoplasm Area' | *'JPH2'* | -0.70 | 0.00165 | Average |
| 'Nuclear/Cytoplasm Area' | *'KIFC3'* | -0.73 | 0.00078 | Average |
| 'Nuclear/Cytoplasm Area' | *'LAMB1'* | -0.75 | 0.00054 | Average |
| 'Nuclear/Cytoplasm Area' | *'MAFF'* | -0.73 | 0.00091 | Average |
| 'Nuclear/Cytoplasm Area' | *'MCAM'* | -0.72 | 0.00105 | Average |
| 'Nuclear/Cytoplasm Area' | *'MCM6'* | 0.75 | 0.00054 | Average |
| 'Nuclear/Cytoplasm Area' | *'MVD'* | 0.74 | 0.00067 | Average |
| 'Nuclear/Cytoplasm Area' | *'NAT15'* | 0.71 | 0.00139 | Average |
| 'Nuclear/Cytoplasm Area' | *'NDEL1'* | -0.77 | 0.00026 | Average |
| 'Nuclear/Cytoplasm Area' | *'NDOR1'* | 0.71 | 0.00145 | Average |
| 'Nuclear/Cytoplasm Area' | *'NMRAL1'* | 0.72 | 0.00100 | Average |
| 'Nuclear/Cytoplasm Area' | *'NOL7'* | -0.77 | 0.00026 | Average |
| 'Nuclear/Cytoplasm Area' | *'NOXA1'* | 0.74 | 0.00067 | Average |
| 'Nuclear/Cytoplasm Area' | *'NUAK1'* | -0.74 | 0.00070 | Average |
| 'Nuclear/Cytoplasm Area' | *'NUBP2'* | 0.80 | 0.00011 | Average |
| 'Nuclear/Cytoplasm Area' | *'PAPLN'* | -0.70 | 0.00152 | Average |
| 'Nuclear/Cytoplasm Area' | *'PIK3CD'* | -0.72 | 0.00121 | Average |
| 'Nuclear/Cytoplasm Area' | *'PMF1'* | 0.72 | 0.00100 | Average |
| 'Nuclear/Cytoplasm Area' | *'PRKAR1B'* | 0.73 | 0.00095 | Average |
| 'Nuclear/Cytoplasm Area' | *'PRR14'* | 0.72 | 0.00105 | Average |
| 'Nuclear/Cytoplasm Area' | *'RAB40C'* | 0.74 | 0.00067 | Average |
| 'Nuclear/Cytoplasm Area' | *'RAG1AP1'* | 0.70 | 0.00152 | Average |
| 'Nuclear/Cytoplasm Area' | *'RHOT1'* | 0.79 | 0.00017 | Average |
| 'Nuclear/Cytoplasm Area' | *'RHOT2'* | 0.77 | 0.00031 | Average |
| 'Nuclear/Cytoplasm Area' | *'RNF145'* | -0.71 | 0.00126 | Average |
| 'Nuclear/Cytoplasm Area' | *'RPL26'* | -0.73 | 0.00082 | Average |
| 'Nuclear/Cytoplasm Area' | *'RSU1'* | -0.79 | 0.00013 | Average |
| 'Nuclear/Cytoplasm Area' | *'SCG2'* | -0.73 | 0.00095 | Average |
| 'Nuclear/Cytoplasm Area' | *'ST5'* | -0.77 | 0.00026 | Average |
| 'Nuclear/Cytoplasm Area' | *'TGFB2'* | -0.72 | 0.00115 | Average |
| 'Nuclear/Cytoplasm Area' | *'VCL'* | -0.74 | 0.00067 | Average |
| 'Nuclear/Cytoplasm Area' | *'VCP'* | 0.72 | 0.00105 | Average |
| 'Nuclear/Cytoplasm Area' | *'WDR40A'* | 0.74 | 0.00070 | Average |
| 'Nuclear/Cytoplasm Area' | *'ZNF263'* | 0.76 | 0.00033 | Average |
| 'Nuclear/Cytoplasm Area' | *'ZNF398'* | 0.71 | 0.00139 | Average |
| 'Nuclear/Cytoplasm Area' | *'ZNF434'* | 0.73 | 0.00091 | Average |
| 'Nuclear/Cytoplasm Area' | *'AGTPBP1'* | -0.79 | 0.00017 | Std |
| 'Nuclear/Cytoplasm Area' | *'BRI3BP'* | 0.71 | 0.00145 | Std |
| 'Nuclear/Cytoplasm Area' | *'HAS2'* | -0.79 | 0.00012 | Std |
| 'Nuclear/Cytoplasm Area' | *'KLHL13'* | 0.74 | 0.00067 | Std |
| 'Nuclear/Cytoplasm Area' | *'NIPA1'* | 0.71 | 0.00145 | Std |
| 'Nuclear/Cytoplasm Area' | *'NUBPL'* | 0.71 | 0.00139 | Std |
| 'Nuclear/Cytoplasm Area' | *'PDCD6'* | 0.70 | 0.00165 | Std |
| 'Nuclear/Cytoplasm Area' | *'PDE7B'* | -0.79 | 0.00013 | Std |
| 'Nuclear/Cytoplasm Area' | *'PDLIM7'* | -0.80 | 0.00011 | Std |
| 'Nuclear/Cytoplasm Area' | *'RGS4'* | -0.79 | 0.00013 | Std |
| 'Nuclear/Cytoplasm Area' | *'RPA3'* | 0.78 | 0.00022 | Std |
| 'Nuclear/Cytoplasm Area' | *'TEX261'* | -0.84 | 0.00000 | Std |
| 'Nuclear/Cytoplasm Area' | *'ZNF280B'* | 0.72 | 0.00100 | Std |
| 'Protrusions Area' | *'AC087742.9'* | 0.71 | 0.00139 | Average |
| 'Protrusions Area' | *'ADAM9'* | 0.73 | 0.00078 | Average |
| 'Protrusions Area' | *'ADM'* | 0.73 | 0.00078 | Average |
| 'Protrusions Area' | *'C9orf23'* | -0.79 | 0.00016 | Average |
| 'Protrusions Area' | *'C9orf23'* | -0.71 | 0.00139 | Average |
| 'Protrusions Area' | *'C9orf86'* | -0.71 | 0.00139 | Average |
| 'Protrusions Area' | *'COMMD3'* | -0.73 | 0.00078 | Average |
| 'Protrusions Area' | *'COX7B2'* | 0.75 | 0.00057 | Average |
| 'Protrusions Area' | *'CTB-54D4.1'* | 0.72 | 0.00105 | Average |
| 'Protrusions Area' | *'EMILIN2'* | 0.74 | 0.00063 | Average |
| 'Protrusions Area' | *'FAM173A'* | -0.71 | 0.00132 | Average |
| 'Protrusions Area' | *'HIRIP3'* | -0.78 | 0.00024 | Average |
| 'Protrusions Area' | *'JPH2'* | 0.77 | 0.00031 | Average |
| 'Protrusions Area' | *'KIAA1467'* | -0.73 | 0.00086 | Average |
| 'Protrusions Area' | *'NME3'* | -0.73 | 0.00078 | Average |
| 'Protrusions Area' | *'PRDX2'* | -0.80 | 0.00009 | Average |
| 'Protrusions Area' | *'RNF145'* | 0.76 | 0.00043 | Average |
| 'Protrusions Area' | *'RP11-122C9.1'* | 0.75 | 0.00054 | Average |
| 'Protrusions Area' | *'TATDN1'* | 0.75 | 0.00054 | Average |
| 'Protrusions Area' | *'TRAM1'* | 0.75 | 0.00051 | Average |
| 'Protrusions Area' | *'VCL'* | 0.72 | 0.00100 | Average |
| 'Protrusions Area' | *'WDR40A'* | -0.74 | 0.00070 | Average |
| 'Protrusions Area' | *'AC087742.9'* | 0.73 | 0.00082 | Std |
| 'Protrusions Area' | *'C9orf23'* | -0.71 | 0.00126 | Std |
| 'Protrusions Area' | *'COL15A1'* | 0.74 | 0.00060 | Std |
| 'Protrusions Area' | *'EAF2'* | -0.71 | 0.00126 | Std |
| 'Protrusions Area' | *'EMILIN2'* | 0.85 | 0.00000 | Std |
| 'Protrusions Area' | *'HIRIP3'* | -0.72 | 0.00100 | Std |
| 'Protrusions Area' | *'NUDT2'* | -0.70 | 0.00152 | Std |
| 'Protrusions Area' | *'RP11-122C9.1'* | 0.73 | 0.00086 | Std |
| 'Protrusions Area' | *'TSHZ1'* | -0.70 | 0.00165 | Std |
| 'Ruffliness' | *'AGTPBP1'* | 0.72 | 0.00100 | Average |
| 'Ruffliness' | *'ARHGEF7'* | 0.70 | 0.00152 | Average |
| 'Ruffliness' | *'C20orf29'* | 0.86 | 0.00000 | Average |
| 'Ruffliness' | *'CALD1'* | 0.71 | 0.00145 | Average |
| 'Ruffliness' | *'CALU'* | 0.73 | 0.00086 | Average |
| 'Ruffliness' | *'CCDC136'* | 0.74 | 0.00070 | Average |
| 'Ruffliness' | *'COL4A2'* | 0.73 | 0.00091 | Average |
| 'Ruffliness' | *'CTNS'* | 0.71 | 0.00139 | Average |
| 'Ruffliness' | *'EMILIN2'* | 0.77 | 0.00031 | Average |
| 'Ruffliness' | *'ETFB'* | -0.71 | 0.00145 | Average |
| 'Ruffliness' | *'FUK'* | -0.71 | 0.00145 | Average |
| 'Ruffliness' | *'GMPR'* | 0.72 | 0.00110 | Average |
| 'Ruffliness' | *'HSD17B7P2'* | -0.77 | 0.00026 | Average |
| 'Ruffliness' | *'ITGB1BP1'* | 0.71 | 0.00132 | Average |
| 'Ruffliness' | *'OSBPL7'* | -0.77 | 0.00026 | Average |
| 'Ruffliness' | *'PDE7B'* | 0.78 | 0.00018 | Average |
| 'Ruffliness' | *'PDLIM7'* | 0.77 | 0.00026 | Average |
| 'Ruffliness' | *'PIK3CD'* | 0.70 | 0.00165 | Average |
| 'Ruffliness' | *'RANGRF'* | 0.79 | 0.00012 | Average |
| 'Ruffliness' | *'RAX2'* | 0.71 | 0.00132 | Average |
| 'Ruffliness' | *'RHOT1'* | -0.70 | 0.00158 | Average |
| 'Ruffliness' | *'RNASEK'* | 0.70 | 0.00152 | Average |
| 'Ruffliness' | *'TEX261'* | 0.73 | 0.00078 | Average |
| 'Ruffliness' | *'ZNF280B'* | -0.70 | 0.00158 | Average |
| 'Ruffliness' | *'ARG2'* | -0.72 | 0.00121 | Std |
| 'Ruffliness' | *'ARHGEF7'* | 0.72 | 0.00115 | Std |
| 'Ruffliness' | *'C13orf8'* | 0.73 | 0.00086 | Std |
| 'Ruffliness' | *'C20orf29'* | 0.86 | 0.00000 | Std |
| 'Ruffliness' | *'CARKD'* | 0.73 | 0.00091 | Std |
| 'Ruffliness' | *'CTNS'* | 0.80 | 0.00009 | Std |
| 'Ruffliness' | *'CUL4A'* | 0.71 | 0.00132 | Std |
| 'Ruffliness' | *'H2AFZ'* | -0.72 | 0.00115 | Std |
| 'Ruffliness' | *'ITGB1BP1'* | 0.88 | 0.00000 | Std |
| 'Ruffliness' | *'MRE11A'* | -0.74 | 0.00074 | Std |
| 'Ruffliness' | *'TMEM189-UBE2V1'* | 0.72 | 0.00110 | Std |
| 'Ruffliness' | *'ZNF343'* | 0.78 | 0.00020 | Std |

Supplemental Table S3: Shape-correlated genes enrichment for GO ontology and KEGG pathways

| **Selected enrichment of shape correlated genes for GO processes** | | | |
| --- | --- | --- | --- |
| **GO_id** | **Term** | **Number Of Genes** | **FDR p-value** |
| GO:0048513 | organ development | 69 | 0.00429 |
| GO:0009888 | tissue development | 55 | 0.0000357 |
| GO:0060429 | epithelium development | 43 | 0.0000357 |
| GO:0060485 | mesenchyme development | 12 | 0.00446 |
| GO:0022603 | regulation of anatomical structure morphogenesis | 27 | 0.00846 |
| GO:0048729 | tissue morphogenesis | 25 | 0.00103 |
| GO:0002009 | morphogenesis of an epithelium | 20 | 0.00323 |
| GO:0000902 | **cell morphogenesis** | 32 | 0.00275 |
| GO:2000145 | **regulation of cell motility** | 26 | 0.000623 |
| GO:0030154 | **cell differentiation** | 77 | 0.00188 |
| GO:0010718 | positive regulation of epithelial to mesenchymal transition | 6 | 0.00202 |
| GO:0007155 | **cell adhesion** | 28 | 0.021 |
| GO:0030198 | **extracellular matrix organization** | 25 | 0.0000357 |
| GO:0034330 | cell junction organization | 13 | 0.00518 |
| GO:0030030 | **cell projection organization** | 30 | 0.0219 |
| GO:0010628 | positive regulation of gene expression | 37 | 0.0414 |
| GO:0034405 | response to fluid shear stress | 5 | 0.0142 |
| GO:0006810 | transport | 73 | 0.0334 |
| **Enrichment of shape-correlated genes for KEGG pathways** | | | |
| **GO_id** | **Term** | **Number Of Genes** | **FDR p-value** |
| hsa05146 | Amoebiasis | 10 | 0.00258 |
| hsa04512 | **ECM-receptor interaction** | 9 | 0.00258 |
| hsa04510 | **Focal adhesion** | 13 | 0.00421 |
| hsa05200 | **Pathways in cancer** | 17 | 0.00421 |
| hsa05222 | Small cell lung cancer | 8 | 0.00684 |

Supplementary Table S4: Enrichment of genes correlated with individual shape features for GO Ontology and KEGG pathways

| **Enrichment of genes correlated with Cell width/Cell length** | | | | |
| --- | --- | --- | --- | --- |
| **Category** | **Term** | **Genes** | **FDR p-value** |  |
| GOTERM_CC_FAT | GO:0005856~ CYTOSKELETON | *ANLN, KIFC3, MAP1B, GYPC, SPTBN1, SLC9A3R1* | 1.82E-02 |  |
| GOTERM_BP_FAT | GO:0042127~ Regulation of cell proliferation | *CCDC88A, CD47, EMP3, ETS1, FABP7, HDAC9, INHA, NRP1* | 3.45E-4 |  |
| GOTERM_BP_FAT | GO:0007155~ cell adhesion | *COL18A1, CD47, NRP1, LYPD3, PVRL1, ATP2C1, ICAM3, CNTNAP3B, COL5A1* | 4.09E-2 |  |
| **Enrichment of genes correlated with Nuclear Roundness** | | | |  |
| **Category** | **Term** | **Genes** | **FDR pvalue** |  |
| GOTERM_CC_FAT | GO:0005739~ mitochondrion | *GLT8D1, IMMP1L, ISCU, MRPS26, TRMU, MRPL12, MRPS18A, NDUFA9, IDH3B, TMEM70* | 1.17E-03 |  |
| **Enrichment of genes correlated with Nucleus area/Cell area** | | | |  |
| **Category** | **Term** | **Genes** | **FDR pvalue** |  |
| KEGG_PATHWAY | hsa04510: Focal adhesions | *COL4A2, COL4A1, PIK3CD, COL6A2, ITGA1, LAMB1, VCL* | 1.02E-07 |  |
| GOTERM_CC_FAT | GO:0031012 extracellular matrix | *COL4A2, HAPLN3, COL4A1, COL6A2, PAPLN, LAMB1, EMILIN2, TGFB2* | 5.29E-07 |  |

Supplemental Table S5: The interaction between the proteins encoded by shape-correlated genes and the selected TFs

| **TF** | **Interact with** |
| --- | --- |
| KLF4 | MED8, SEC11C |
| MYC | CUL4A, ITGB1, RAGE, PAM |
| RELA | AR, ARHGEF7, HMGA2, MAP2K6, RAC3, SCG2, |
| SMAD2 | HMGA2 |
| SMAD3 | AR, ETS1, HMGA2, JPH3, LMNA, ODC1, PRKAR1B, SPTBN1 |
| SNAI1 | No direct interaction |
| SNAI2 | No direct interaction |
| SOX2 | No direct interaction |
| TAZ | No direct interaction |
| TWIST1 | No direct interaction |
| ZEB1 | No direct interaction |
| ZEB2 | No direct interaction |
| YAP1 | SLC9A3R1 |

**Supplemental Table S6: Direct paths between shape features and SMAD3, RELA, or YAP1 in the shape-gene network.**

| **Morphological feature (source)** | **TF (target)** | **Protein interactions involved in each path from source to target** |
| --- | --- | --- |
| N/C area | RELA | 1) AR  2) SCG2  3) HMGA2 |
| N/C area | YAP1 | No direct path |
| N/C area | SMAD3 | 1) AR  2) PRKAR1B  3) HMGA2 |
| Cell W/L | RELA | 1) TRIO -> RAC3  2) TRIO -> RAC3-> AR  3) DEPDC1 -> AR  4) GAK -> AR  5) FAM174B -> AR  6) ETS1 -> AR |
| Cell W/L | YAP1 | 1) SLC9A3R1 |
| Cell W/L | SMAD3 | 1) ETS1  2) SPTBN1  3) ODC1 |
| NF | RELA and YAP1 | No direct path |
| NF | SMAD3 | 1) CD151 -> ITGB1 -> LMNA  2) CD151 -> ITGB1 -> LMNA -> PRKAR1B |
| Nuclear roundness | RELA | 1) MAP2K6  2) RAC3 |
| Nuclear roundness | YAP1 | No direct path |
| Nuclear roundness | SMAD3 | 1) NARF -> LMNA  2) MRPS26 -> LMNA  3) RAC3 -> AR |
| Ruffliness | RELA | 1) PIK3CD -> RAC3  2) ARHGEF7 |
| Ruffliness | YAP1 | No direct path |
| Ruffliness | SMAD3 | 1) ARG2 ->ODC1 |
| Protrusion area | RELA | No direct path |
| Protrusion area | YAP1 | No direct path |
| Protrusion area | SMAD3 | 1) VCL -> PTK2 ->SPTBN1  2) EMILIN2 -> CSF1R -> ETS1-> AR |
| Nuclear area | RELA | No direct path |
| Nuclear area | YAP1 | No direct path |
| Nuclear area | SMAD3 | 1) ARG2 ->ODC1 |
| Centres distance | RELA | No direct path |
| Centres distance | YAP1 | No direct path |
| Centres distance | SMAD3 | 1) JPH3  2) TNS3 -> PTK2 -> SPTBN1 |
| Area | RELA | 1) RAD9A -> AR  2) PLXNA1 -> RAC3 |
| Area | YAP1 | No direct path |
| Area | SMAD3 | 1) PTK2 -> SPTBN1  2) ITGB1 -> PRKAR1B  3) RAD9A -> AR |
| Nuclear W/L | RELA | 1) DPYSL4 -> PLXNA1 -> RAC3 |
| Nuclear W/L | YAP1 | No direct path |
| Nuclear W/L | SMAD3 | 1) LMNA  2) CTTN -> PTK2 -> SPTBN1 |

Supplemental Table S7: TF activation and expression data as in Fig. 4. Number of overlapping target genes is the number of genes that change significantly after the depletion of a shape-correlated genes or a TF.

| **Shape correlated gene** | **TF** | **Number of overlapping target genes** | **Overlapping percentage** | **P-value** | **Affects expression of TF** |
| --- | --- | --- | --- | --- | --- |
| *RAD9A* | *RELA* | 167 | 0.27 | 0.0064 | No |
| *LMNA* | *RELA* | 110 | 0.18 | 0.0001 | No |
| *TNS3* | *RELA* | 118 | 0.19 | 0.0199 | Yes |
| *TRIO* | *RELA* | 112 | 0.18 | 0.0106 | No |
| *PLXNA1* | *RELA* | 102 | 0.16 | 0.0022 | No |
| *SLC9A3R1* | *RELA* | 139 | 0.22 | 0.0000 | Yes |
| *HMGA2* | *RELA* | 112 | 0.18 | 0.0414 | No |
| *ODC1* | *RELA* | 108 | 0.17 | 0.0405 | Yes |
| *GAK* | *RELA* | 95 | 0.15 | 0.0458 | No |
| *MAP2K6* | *RELA* | 98 | 0.16 | 0.0351 | No |
| *FAM173A* | *RELA* | 76 | 0.12 | 0.0537 | No |
| *ARHGEF7* | *RELA* | 69 | 0.11 | 0.0369 | No |
| *NARF* | *RELA* | 71 | 0.11 | 0.0483 | No |
| *SCG2* | *RELA* | 62 | 0.10 | 0.0532 | No |
| *PTK2* | *RELA* | 58 | 0.09 | 0.0283 | Yes |
| *DEPDC1* | *RELA* | 156 | 0.25 | 0.0282 | Yes |
| *DEPDC1* | *SMAD3* | 164 | 0.30 | 0.0045 | Yes |
| *TRIO* | *SMAD3* | 134 | 0.25 | 0.0000 | No |
| *TNS3* | *SMAD3* | 130 | 0.24 | 0.0000 | No |
| *SLC9A3R1* | *SMAD3* | 156 | 0.29 | 0.0317 | No |
| *LMNA* | *SMAD3* | 104 | 0.19 | 0.0000 | No |
| *PLXNA1* | *SMAD3* | 100 | 0.18 | 0.0001 | Yes |
| *RAD9A* | *SMAD3* | 113 | 0.21 | 0.0076 | No |
| *HMGA2* | *SMAD3* | 97 | 0.18 | 0.0417 | No |
| *MAP2K6* | *SMAD3* | 87 | 0.16 | 0.0427 | No |
| *ODC1* | *SMAD3* | 87 | 0.16 | 0.0181 | No |
| *FAM173A* | *SMAD3* | 73 | 0.13 | 0.0404 | No |
| *GAK* | *SMAD3* | 78 | 0.14 | 0.0505 | No |
| *PTK2* | *SMAD3* | 62 | 0.11 | 0.0500 | No |
