# Supplementary material for: Identification of clinically predictive metagenes that encode components of a network coupling cell shape to transcription by image-omics
Source: Genome Res. 2017 Feb;27(2):196–207. doi: 10.1101/gr.202028.115 (PMC5287226; doi:10.1101/gr.202028.115)
Supplement: Supplemental Material [file supp_gr.202028.115_Supplemental_Table_S8-S11.docx]

**Supplemental Tables S8-S11**

**Supplemental Table S8:** **Metagene models (mean).** Multilinear regression models of morphological features average. Coefficients reflect the weight of each gene in the feature model.

| **Cell area** | |
| --- | --- |
| **Gene** | **Coefficient** |
| 'constant' | 1.41117375 |
| *'HES4'* | -0.002734225 |
| *'PLXNA1'* | 0.196951987 |
| *'ATP2C1'* | -0.345024186 |
| *'PAM'* | 0.128184741 |
| *'SYT12'* | 0.329365022 |
| *'RAD9A'* | -0.313649231 |
| *'NFE2L1'* | 0.171292798 |
| *'IDH3G'* | -0.241262418 |
| **R^2^** | 0.918757754 |
| **Error** | 0.013085057 |
|  | |
| **Protrusions area** | |
| **Gene** | **Coefficient** |
| 'constant' | 0.461322513 |
| *'TATDN1'* | 0.079789924 |
| *'WDR40A'* | 0.18536143 |
| *'C9orf23'* | -0.430494566 |
| *'COMMD3'* | -0.189134864 |
| *'AC087742.9'* | 0.300917687 |
| *'EMILIN2'* | 0.07216938 |
| *'PRDX2'* | 0.004266063 |
| **R^2^** | 0.92202763 |
| **Error** | 0.013371377 |
|  | |
| **Ruffliness** | |
| **Gene** | **Coefficient** |
| 'constant' | 0.315063085 |
| *'TEX261'* | 0.323143262 |
| *'OSBPL7'* | -0.289724794 |
| *'EMILIN2'* | 0.077490297 |
| *'ETFB'* | -0.134176222 |
| **R^2^** | 0.94073618 |
| **Error** | 0.009166668 |
|  | |
| **Centres Distance** | |
| **Gene** | **Coefficient** |
| 'constant' | 0.503612649 |
| *'C6orf64'* | -0.179848053 |
| *'ADM'* | 0.056629136 |
| *'N4BP2L2'* | -0.123335628 |
| *'UBE2G2'* | 0.188374254 |
| **R^2^** | 0.909391063 |
| **Error** | 0.006452909 |
|  | |
| **Neighbour Fraction** | |
| **Gene** | **Coefficient** |
| 'constant' | -0.935485188 |
| *'AGTPBP1'* | -0.218269775 |
| *'AARS'* | 0.220209656 |
| *'C20orf29'* | -0.21530821 |
| *'ZNF280B'* | 0.117365629 |
| *'KLHL13'* | 0.229378831 |
| **R^2^** | 0.907866899 |
| **Error** | 0.013696057 |
|  |  |
| **Nuclear/Cytoplasm area** | |
| **Gene** | **Coefficient** |
| 'constant' | 0.265350643 |
| *'SCG2'* | -0.054315738 |
| *'LAMB1'* | -0.057115336 |
| *'NUAK1'* | -0.039093833 |
| *'AR'* | 0.185814547 |
| **R^2^** | 0.92789066 |
| **Error** | 0.005448745 |
|  | |
| **Cell Width/Length** | |
| **Gene** | **Coefficient** |
| 'constant' | -0.601873049 |
| *'CRIM1'* | 0.159345798 |
| *'ABCA12'* | -0.926487519 |
| *'CD47'* | 0.105530023 |
| *'LEPREL1'* | 0.068533488 |
| *'SLC46A2'* | 0.449839139 |
| *'TMEM205'* | 0.206823929 |
| **R^2^** | 0.956200413 |
| **Error** | 0.003282531 |
|  |  |
| **Nucleus area** | |
| **Gene** | **Coefficient** |
| 'constant' | -1.982559098 |
| *'SCCPDH'* | -0.079171397 |
| *'C2orf82'* | -0.246761932 |
| *'OTUD6B'* | 0.125499375 |
| *'UTP23'* | 0.063215567 |
| *'AC087742.9'* | 0.27418185 |
| *'UBE2G2'* | 0.078807449 |
| **R^2^** | 0.776786859 |
| **Error** | 0.035223234 |
|  | |
| **Nuclear roundness** | |
| **Gene** | **Coefficient** |
| 'constant' | 1.096849858 |
| *'CCDC53'* | 0.127794394 |
| *'ALDH1L2'* | 0.082140241 |
| *'TOX3'* | -0.180806042 |
| *'CTA-221G9.5'* | -0.11992781 |
| **R^2^** | 0.933239115 |
| **Error** | 0.006912405 |
|  | |
| **Nuclear width/length** | |
| **Gene** | **Coefficient** |
| 'constant' | -1.633970693 |
| *'DDX59'* | -0.208236688 |
| *'PAICS'* | 0.110501958 |
| *'NUP188'* | 0.116883642 |
| *'PSMB5'* | 0.173781986 |
| *'ENO3'* | -0.107434052 |
| *'MED25'* | 0.058187673 |
| **R^2^** | 0.912524389 |
| **Error** | 0.009526329 |

**Supplemental Table S9: Metagene models (std).** Multilinear regression models of morphological features std. Coefficients reflect the weight of each gene in the feature model.

| **Cell Area std** | |
| --- | --- |
| **Gene** | **Coefficient** |
| 'constant' | 1.207454141 |
| *'CHRAC1'* | -0.227520138 |
| *'LARP5'* | 0.248216157 |
| *'CHST15'* | -0.148591204 |
| **R^2^** | 0.009500905 |
| **Error** | 0.854953899 |
|  | |
| **Protrusions area std** | |
| **Gene** | **Coefficient** |
| 'constant' | -1.421351151 |
| *'NUDT2'* | -0.023136093 |
| *'C9orf23'* | -0.142397752 |
| *'COL15A1'* | 0.176293984 |
| *'HIRIP3'* | -0.051825689 |
| *'AC087742.9'* | 0.394272087 |
| *'EMILIN2'* | 0.016541225 |
| *'TSHZ1'* | -0.12856542 |
| **R^2^** | 0.863419718 |
| **Error** | 0.020214347 |
|  | |
| **Ruffliness std** | |
| **Gene** | **Coefficient** |
| 'constant' | 2.706988455 |
| *'ITGB1BP1'* | -0.043589602 |
| *'H2AFZ'* | -0.12542915 |
| *'MRE11A'* | -0.086779264 |
| *'CARKD'* | -0.024384356 |
| *'ARHGEF7'* | -0.118788917 |
| *'C13orf8'* | 0.127260389 |
| *'ARG2'* | -0.307741978 |
| *'CTNS'* | -0.077962208 |
| *'ZNF343'* | -0.053447798 |
| *C20orf29'* | 0.471753199 |
| **R^2^** | 0.811193333 |
| **Error** | 0.023291235 |
|  | |
| **Centres Distance std** | |
| **Gene** | **Coefficient** |
| 'constant' | -0.489126145 |
| *'FAM183A'* | 0.119866299 |
| *'LRRC24'* | -0.049033857 |
| *'AC011511.11'* | 0.100181031 |
| *'UBE2G2'* | 0.140067836 |
| *'RAB9B'* | -0.233817764 |
| **R^2^** | 0.826261485 |
| **Error** | 0.01206078 |
|  | |
| **Neighbour Fraction std** | |
| **Gene** | **Coefficient** |
| 'constant' | -0.236884091 |
| *'GSTM1'* | -0.062297198 |
| *'C2orf49'* | 0.145977062 |
| *'WNT7A'* | 0.17800167 |
| *'DEAF1'* | -0.154592324 |
| *'C11orf87'* | -0.077252637 |
| *'BTBD11'* | 0.057783462 |
| *'KLK7'* | -0.0144777 |
| **R^2^** | 0.90182921 |
| **Error** | 0.010402739 |
|  | |
| **Nuclear/Cytoplam Area std** | |
| **Gene** | **Coefficient** |
| 'constant' | -0.160770683 |
| *'RGS4'* | -0.11371112 |
| *'PDCD6'* | -0.120041744 |
| *'PDLIM7'* | -0.077415554 |
| *'RPA3'* | 0.193005393 |
| *'HAS2'* | -0.15825368 |
| *'BRI3BP'* | -0.115453331 |
| *'NUBPL'* | 0.220827419 |
| *'NIPA1'* | 0.180401509 |
| **R^2^** | 0.905554315 |
| **Error** | 0.01279018 |
|  | |
| **Cell Width/Length std** | |
| **Gene** | **Coefficient** |
| 'constant' | 0.348607622 |
| *'TMEM79'* | -0.177878419 |
| *'SRI'* | 0.105365138 |
| *'ATP6V1G1'* | 0.229367899 |
| *'COL5A1'* | 0.049755281 |
| *'NHLRC3'* | -0.268550437 |
| **R^2^** | 0.916819247 |
| **Error** | 0.007217092 |
|  | |
| **Nucleus area std** | |
| **Gene** | **Coefficient** |
| 'constant' | -4.726756723 |
| *'ANKRD50'* | -0.15059582 |
| *'MFAP3L'* | 0.230084312 |
| *'C9orf82'* | -0.160513529 |
| *'FAM25A'* | 0.116438513 |
| *'OLR1'* | -0.041766766 |
| *'CNTN1'* | 0.089049629 |
| *'ARG2'* | 0.252398965 |
| *'NSFL1C'* | 0.27359929 |
| *'IDH3B'* | 0.072661551 |
| **R^2^** | 0.880477366 |
| **Error** | 0.020717323 |
|  | |
| **Nuclear roundness std** | |
| **Gene** | **Coefficient** |
| 'constant' | 0.735978949 |
| *'MRPS18A'* | 0.333925139 |
| *'ISCU'* | -0.257055414 |
| *'MRPL12'* | 0.112304036 |
| *'RAC3'* | -0.14453669 |
| *'ANKRD24'* | -0.258329336 |
| *'ILVBL'* | 0.068219789 |
| **R^2^** | 0.916048342 |
| **Error** | 0.009684851 |
|  | |
| **Nuclear Width/Length std** | |
| **Gene** | **Coefficient** |
| 'constant' | -0.083762789 |
| *'CLCN6'* | -0.049179347 |
| *'BET1'* | -0.29073928 |
| *'SLC2A8'* | 0.13611462 |
| *'PSME1'* | 0.136631616 |
| *'TOX3'* | 0.071697111 |
| **R^2^** | 0.907211468 |
| **Error** | 0.01073671 |

**Supplemental Table S10:** **Cox-regression results.** Cox-regression univariate and multivariate analysis to model morphological metagene effects on the survival of the patients in METABRIC dataset. Effects are tested for metagene only, metagene + size +node, and metagene + size + lymph node + grade. P-values are based on the Wald statistics. HR: Hazard ratio, CI: 95% confidence interval.

| **Metagene** | **Variable** | **Discovery set** | | **Validation set** | |
| --- | --- | --- | --- | --- | --- |
|  |  | **HR (CI)** | ***P*-value** | **HR (CI)** | ***P*-value** |
| **Cell area** | Uni- | 0.72 (0.56-0.93) | 0.012 | 0.70 (0.54-0.90) | 0.006 |
|  | Multi- (no grade) | 0.76 (0.59-0.98) | 0.033 | 0.81 (0.62-1.05) | .11 |
|  | Multi- (with grade) | 0.84 (0.65-1.08) | 0.17 | 0.91 (0.70-1.19) | 0.51 |
|  | Multi-size | 1.23 (0.94-1.61) | 0.12 | 1.97 (1.49-2.61) | 2.45E-06 |
|  | Multi-node | 2.31 (1.77-3.02) | 8.75E-10 | 2.15 (1.65-2.82) | 2.35E-08 |
|  | Multi-grade | 1.71 (1.35-2.15) | 6.86E-06 | 1.44 (1.16-1.79) | 0.0008 |
| **Cell width/length** | Uni- | 0.60 (0.47-.77) | 5.87E-05 | 0.64 (0.50-0.82) | 5.87E-05 |
|  | Multi- (no grade) | 0.69 (0.54-0.89) | 0.003 | 0.73 (0.56-0.93) | 0.013 |
|  | Multi- (with grade) | 0.78 (0.61-1.01) | 0.06 | 0.85 (0.65-1.11) | 0.23 |
|  | Multi-size | 1.22 (0.93-1.59) | 0.15 | 1.96 (1.48-2.60) | 2.76E-06 |
|  | Multi-node | 2.29 (1.75-2.99) | 1.48E-09 | 2.15 (1.64-2.82) | 2.40E-08 |
|  | Multi-grade | 1.67 (1.32-2.11) | 1.76E-05 | 1.42 (1.14-1.76) | 0.00178606 |
| **Neighbour fraction std** | Uni- | 2.08 (1.60-2.71) | 5.28E-08 | 1.48 (1.12-1.95) | 0.006 |
|  | Multi- (no grade) | 1.91 (1.47-2.49) | 1.79E-06 | 1.34 (1.01-1.78) | 0.04 |
|  | Multi- (with grade) | 1.76 (1.34-2.30) | 3.54E-05 | 1.13 (0.84-1.51) | 0.43 |
|  | Multi-size | 1.21 (0.93-1.58) | 0.16 | 1.99 (1.50-2.63) | 1.69E-06 |
|  | Multi-node | 2.30 (1.75-3.00) | 1.30E-09 | 2.16 (1.65-2.82) | 2.15E-08 |
|  | Multi-grade | 1.64 (1.31-2.07) | 2.21E-05 | 1.44 (1.16-1.78) | 0.001 |
| **Cell area std** | Uni- | 1.54 (1.18-1.99) | 0.001 | 1.54 (1.17-2.00) | 0.002 |
|  | Multi- (no grade) | 1.48 (1.14-1.93) | 0.003 | 1.60 (1.22-2.10) | 0.0006 |
|  | Multi- (with grade) | 1.32 (1.01-1.72) | 0.04 | 1.49 (1.12-1.97) | 0.005 |
|  | Multi-size | 1.25 (0.96-1.63) | 0.10 | 1.97 (1.49-2.62) | 2.11E-06 |
|  | Multi-node | 2.31 (1.77-3.03) | 8.14E-10 | 2.22 (1.69-2.90) | 7.59E-09 |
|  | Multi-grade | 1.66 (1.31-2.10) | 2.08E-05 | 1.40 (1.13-1.73) | 0.002 |

**Supplemental Table S11:** metagene model of NF-kB response

| **NF-kB response** | |
| --- | --- |
| **Gene** | **Coefs** |
| 'constant' | -10.42055031 |
| *'DEPDC1'* | -0.121538896 |
| *'ODC1'* | 0.379252787 |
| *'GAK'* | 0.317191036 |
| *'TRIO'* | -0.163350106 |
| *'CSF1R'* | -0.051310313 |
| *'TNS3'* | 0.400096634 |
| *'HMGA2'* | 0.232101022 |
| *'MAP2K6'* | -0.178376316 |
| *'SLC9A3R1'* | 0.146951467 |
| **R^2^** | 0.89568907 |
| **Error** | 0.014908876 |
